# The Use of Transposon Insertion Sequencing to Interrogate the Core Functional Genome of the Legume Symbiont *Rhizobium leguminosarum*

**DOI:** 10.3389/fmicb.2016.01873

**Published:** 2016-11-22

**Authors:** Benjamin J. Perry, Mir S. Akter, Christopher K. Yost

**Affiliations:** Department of Biology, University of ReginaRegina, SK, Canada

**Keywords:** Rhizobium, INSeq, Tn-Seq, core functional genome, metabolism, mannitol

## Abstract

The free-living legume symbiont *Rhizobium leguminosarum* is of significant economic value because of its ability to provide fixed nitrogen to globally important leguminous food crops, such as peas and lentils. Discovery based research into the genetics and physiology of *R. leguminosarum* provides the foundational knowledge necessary for understanding the bacterium's complex lifestyle, necessary for augmenting its use in an agricultural setting. Transposon insertion sequencing (INSeq) facilitates high-throughput forward genetic screening at a genomic scale to identify individual genes required for growth in a specific environment. In this study we applied INSeq to screen the genome of *R. leguminosarum* bv. *viciae* strain 3841 (RLV3841) for genes required for growth on minimal mannitol containing medium. Results from this study were contrasted with a prior INSeq experiment screened on peptide rich media to identify a common set of functional genes necessary for basic physiology. Contrasting the two growth conditions indicated that approximately 10% of the chromosome was required for growth, under both growth conditions. Specific genes that were essential to singular growth conditions were also identified. Data from INSeq screening on mannitol as a sole carbon source were used to reconstruct a metabolic map summarizing growth impaired phenotypes observed in the Embden-Meyerhof-Parnas pathway, Entner-Doudoroff pathway, pentose phosphate pathway, and tricarboxylic acid cycle. This revealed the presence of mannitol dependent and independent metabolic pathways required for growth, along with identifying metabolic steps with isozymes or possible carbon flux by-passes. Additionally, genes were identified on plasmids pRL11 and pRL12 that are likely to encode functional activities important to the central physiology of RLV3841.

## Introduction

*Rhizobium leguminosarum* is a Gram-negative soil and rhizosphere colonizing bacterium that is also capable of engaging in endosymbiosis with specific leguminous plant genera. The host specificity of rhizobial infection is dependent upon the exchange of specific chemical signals between the infecting bacterium and host plant (Oldroyd et al., 2011), and *R. leguminosarum* is often divided into biovars based on infectious host range. The biovar *viciae* is indicative of *Rhizobia* capable of infecting leguminous plants such as peas (*Pisum sativum*), lentils (*Lens culinaris*), and vetch (*Vicia* spp.). When in the endosymbiotic state, *Rhizobium* bacteroids reduce atmospheric nitrogen N_2_ to ammonia, which is then exported to the plant for assimilation. In return, the plant host provides fixed carbon and other micro-nutrients to the bacteroids to sustain the symbiosis (Wielbo, 2012; Udvardi and Poole, 2013). The availability of symbiotically supplied nitrogen enables leguminous plants to satisfy their high nitrogen demands and, in part, contributed to the evolutionary success and diversification of the *Leguminosae* (Doyle and Luckow, 2003).

*R. leguminosarum* bv. *viciae* 3841(RLV3841) (Johnston and Beringer, 1975), has long been considered a model organism for *Rhizobium* research and was one of the first rhizobial strains with a published complete genome sequence (Young et al., 2006). Aside from the overarching agricultural context of studying RLV3841, the model organism provides other interesting avenues of research because of its complex genomic structure (Young et al., 2006) and versatility of physiology and lifestyle. RLV3841 has a relatively large bacterial genome comprised of a single 5.1 Mb chromosome and 6 large, stably maintained, plasmids ranging in size from 0.87 to 0.15 Mb. The RLV3841 genome is predicted to contain approximately 7346 genes, a substantial percentage of these genes (25.2%) are annotated as hypothetical genes of unknown function, warranting further investigation. The use of high-throughput experimental approaches may allow prioritization of the study of individual genes within this large functionally unknown group.

The development of next-generation sequencing technologies has resulted in high-throughput methods of transposon (Tn) mutagenesis to study gene function at a genome scale (Gawronski et al., 2009; Goodman et al., 2009; Langridge et al., 2009; van Opijnen et al., 2009). For example, INSeq was developed through the introduction of a type II restriction enzyme site within the IS element of the himar1C9 *mariner* Tn allowing for the specific capture and PCR amplification of genomic DNA adjacent to the Tn insertion site (Goodman et al., 2011). Next generation sequencing of PCR amplicons derived from DNA isolated from these Tn-mutant libraries allows for the sequencing of millions of Tn insertion tags which can be mapped to the genome sequence and used to enumerate the relative abundance of individual Tn mutants within a mutant population (Barquist et al., 2013; van Opijnen and Camilli, 2013). INSeq and similar high-throughput Tn mutagenesis methods have been used to study the genetic basis of bacterial physiology (Griffin et al., 2011; Brutinel and Gralnick, 2012; Kuehl et al., 2014; Yang et al., 2014; Le Breton et al., 2015; Lee et al., 2015; Meeske et al., 2015; Pechter et al., 2015; Rubin et al., 2015; Hooven et al., 2016; Troy et al., 2016), bacterial resistance to biotic and abiotic factors (Gallagher et al., 2011; Khatiwara et al., 2012; Phan et al., 2013; Byrne et al., 2014; Murray et al., 2015; Shan et al., 2015; Yung et al., 2015; Tran et al., 2016), and colonization of hosts or specific environments (Gawronski et al., 2009; Dong et al., 2013; Kamp et al., 2013; Skurnik et al., 2013; Bishop et al., 2014; Johnson et al., 2014; Verhagen et al., 2014; Wang et al., 2014; Gutierrez et al., 2015; Moule et al., 2015; Turner et al., 2015; Capel et al., 2016). Recently, INSeq was adapted for use in the *Rhizobiaceae*, and was demonstrated to be a suitable tool for high-throughput functional genomic screening in RLV3841 (Perry and Yost, 2014).

In this paper we used INSeq to define a core functional genome (CFG) of RLV3841 and deconstruct central carbon metabolism for growth on mannitol, a preferred carbon source of rhizobia (Vincent, 1970; Geddes and Oresnik, 2014). Comparing the genes required for growth on mannitol with those required for growth on tryptone-yeast extract media, we estimated a core set of functional genes required for optimal growth. Furthermore, the results of this study demonstrate that using INSeq and growth on minimal media is an effective approach to gain new insight into central carbon metabolism in RLV3841.

## Materials and methods

### Growth conditions, strains, and plasmids

*R. leguminosarum* bv. *viciae* 3841 (Johnston and Beringer, 1975) was routinely grown at 30°C using tryptone-yeast (TY) extract growth medium (5.0 g tryptone, 3.0 g yeast extract, 3.5 mM CaCl_2_ per liter H_2_O; TY) or Vincent's minimal medium (VMM) (1.0 g K_2_HPO_4_, 1.0 g KH_2_PO_4_, 0.01 g FeCl_2_6H_2_O, 0.25 g MgSO_4_7H_2_O, 0.1 g CaCl_2_6H_2_O, 0.6 g KNO_3_, 0.1 mg biotin, 0.1 mg Ca-panthenoate, and 0.1 mg thiamine per liter H_2_O) supplemented with 1.% (w/v) mannitol as a carbon source (Vincent, 1970). The donor *E. coli* strain SM10λpir harboring pSAM_Rl was routinely grown on LB medium at 37°C. Antibiotic concentrations were 500 μg/mL streptomycin (Str) and 50 μg/mL neomycin (Neo) for RLV3841, and 100 μg/mL ampicillin (Amp) and 50 μg/mL kanamycin (Kan) for *E. coli*.

### *Mariner* transposon mutant pool generation and mutant selection

Mutant pools were generated as described in Perry and Yost (2014) with minor modifications. Briefly, RLV3841 and *E. coli* SM10λ*pir*(pSAM_Rl) were grown in broth culture until late exponential phase. 1.0 ml of donor and 0.5 ml of recipient strains were mixed in a 1.5 mL microcentrifuge tube and pelleted at 12, 000 g for 3 min. The cell mixture was then washed twice with 1000 μL 1X phosphate buffer saline (PBS), and resuspended in a final volume of approximately 100 μL 1X PBS. Six independent conjugations were spotted onto pre-warmed VMM-mannitol plates and were incubated at 30°C overnight (~18 h). Following incubation each of the 6 conjugation spots were scraped and re-suspended in 1000 μL of 1X PBS and pooled into a total volume of 6 ml, representing the RLV3841 Tn mutant library.

Selection of mutant pools on VMM-mannitol was conducted using six 245 × 245 mm^2^ (Corning) Neo and Str containing VMM-mannitol agar plates. For each selection plate, 500 μL of the RLV3841 mutant pool was spread plated and allowed to dry. The agar plates were incubated at 30 C for 72 hr representing between 15 and 18 generations of RLV3841 growth on minimal media. Cells from each plate were harvested by scraping the thin film of cell growth and re-suspending in 5 mL of 1X PBS, vortexed thoroughly to homogenize the cells, and then a 1000 μL aliquot of each cell suspension was used for cell pelleting and DNA isolation (Perry and Yost, 2014).

### Transposon insertion sequencing

The mutant pools recovered from 2 of 6 selection plates were pooled into 3 independent technical replicates for DNA extraction and Tn insertion sequencing. The method used for library preparation and sequencing is described by Perry and Yost (2014) with modification to the adaptor sequences INSeq_Adpt_Top and INSeq_Adpt_Bottom (Table S1). The final library concentration of the 3 stock library preparations was 1.29, 1.21, and 1.39 ng/μL after size selection. DNA sequencing was performed on an Ion Torrent PGM using 200 bp sequencing chemistry and a 316v2 sequencing chip. The raw sequence output for the 3 technical replicates was 1.4, 1.1, and 1.1 M reads, respectively, and can be found under SRA deposit number: SRR3400585-7 (TY datasets are deposited under SRR3400588-90). Sequencing data from the 3 technical replicates were pooled for a combined total of 3.6 million to achieve sufficient read depth for hidden Markov model analysis (DeJesus and Ioerger, 2013). Raw sequencing data was processed, aligned, and analyzed as previously described (Perry and Yost, 2014). Briefly, raw reads were clipped at the end of the pSAM_Rl *mariner* IR element, and clipped on the 3′ end at the beginning of the INSeq_Adpt sequence. The resulting reads were screened for the presence of a 5′-TA insertion site, and a length ≥15 bp. Trimmed reads were aligned to the RLV3841 reference genome using bowtie, with the option to suppress reads with multiple alignments from the output file enabled. The alignment files were then converted to wig files and analyzed using the tn-hmm.py python module. The pipeline resulted in a total of 2,374,819 reads being aligned onto the RLV3841 reference genome, after quality filtering and discarding of unaligned reads. The HMM then assigned each “TA” insertion site to one of four growth states which was used to assign each gene to a specific growth phenotype (DeJesus and Ioerger, 2013).

### Curation of INSeq data

Outputs from the HMM for both the TY (Perry and Yost, 2014) and VMM dataset were combined based on RLV3841 locus number. Riley functional classifications for each gene were obtained from the lab of Phillip Poole, University of Oxford (http://rhizosphere.org/lab-page/molecular-tools/genomes/rlv3841-genome) and appended to the dataset. Duplicate gene sequences were manually examined using reciprocal BLAST to the RLV3841 reference genome for all genes with <0.30 insertion density to avoid miss-classification as an essential gene due to a lack of mapped insertions as a result of the alignment penalty for multiple mapping locations. The final compiled and curated dataset is found in Supplementary File 1.

### Insertional mutagenesis of RL0920 and RL3335

Two previously uncharacterized VMM-mannitol growth impaired genes (RL0920 and RL3335) were mutated to verify the INSeq growth phenotype data. Mutants were created using a single crossover mutagenesis approach with pJQ200SK (Quandt and Hynes, 1993), as described in Vanderlinde et al. (2010). Briefly, a 563 bp internal fragment of RL0920 was PCR amplified using primers RL0920_Fwd and RL0920_Rev, which introduced 5′ ApaI and 3′ SpeI restriction enzyme sites. The 563 bp amplicon was then directionally cloned into pJQ200SK using ApaI and SpeI. The new vector pJQ200SK-RL0920 was conjugated into RLV3841 wildtype using *E. coli* strain S17-1, and single cross over mutants were selected for on TYSmGm, and then screened for sucrose sensitivity to confirm the plasmid integration. The identical procedure was used to create a single crossover mutant in RL3335 using primers RL3335_Fwd and RL3335_Rev to generate a 603 bp internal gene fragment for cloning into pJQ200SK (Table S1). The resulting mutants in RL0920 and RL3335 were named MA0920 and MA3335.

### Growth curve analysis of MA0920, MA3335, and RLV3841 wildtype

Growth curves of RLV3841, MA0920, and MA3335 were performed using a shaking head Synergy HT Microplate Reader (Biotek) with 250 μL of inoculated growth media per well in a 96-well NuncR Optical Bottom Plate (Thermo Scientific), and a 40 μL Anti-Evaporation Oil (Ibidi) overlay. Inoculated growth medium was prepared by scraping cells from freshly grown TYSm or SmGm plates, washing twice with 1XPBS, standardizing the cell suspension for an approximate initial OD_600_ of 0.01. Cells were grown at 30°C with 10 min of shaking followed by OD_600_ measurements every 30 min, for 72 h. Each growth curve was derived from the mean OD_600_ measurements of 7 replicates. Mean generation times (MGT) were calculated in early exponential phase (OD_600_ < 0.100) and late exponential phase (0.100 < OD_600_ < 0.200) by calculating the average time required to double the optical density of the cell cultures, within the defined growth phases.

## Results

### Tn insertion sequencing and transposition summary of VMM-mannitol mutant pools

The RLV3841 genome contains 140,056 potential mariner insertion sites distributed across the chromosome and 6 mega-plasmids. Insertion densities within the genome ranged from 0.65 to 0.86 across the 7 replicons, with an average insertion density of 0.80 (Table S2). The insertion densities were similar to the insertion densities previously observed in an INSeq experiment using TY medium for selection (Perry and Yost, 2014). HMM analysis of the VMM INSeq data assigned 7.2 and 2.6% of genes to essential (ES) or growth defective (GD) growth phenotypes, respectively (Table S3). Given sufficient generations of growth GD mutants would be excluded from the mutant community. Therefore, ES and GD states were pooled into a single growth impaired (GI) category (9.8% of the genes) for annotation with Riley functional groupings. 87.1% of genes were observed to have no impact on growth (NE), and 1.20% of genes with Tn insertions became over-represented within the mutant communities, and were predicted to confer a growth advantage (GA) phenotype (Table S3). To further simplify interpretation of the INSeq data by focusing exclusively on loss-of-function phenotypes the NE and GA genes were pooled into a single category termed growth neutral (GN). Within the genome 1.6% of genes had sequence duplications resulting in no information concerning their impact on growth due to the multiple Tn insertion tag mapping location penalty imposed. As well, 0.3% of genes were observed to lack a “TA” dinucleotide motif leaving them without a target site for mariner Tn insertion.

### The RLV3841 CFG, VMM-growth impaired, and TY-growth impaired phenotypes

**Figure 1:**
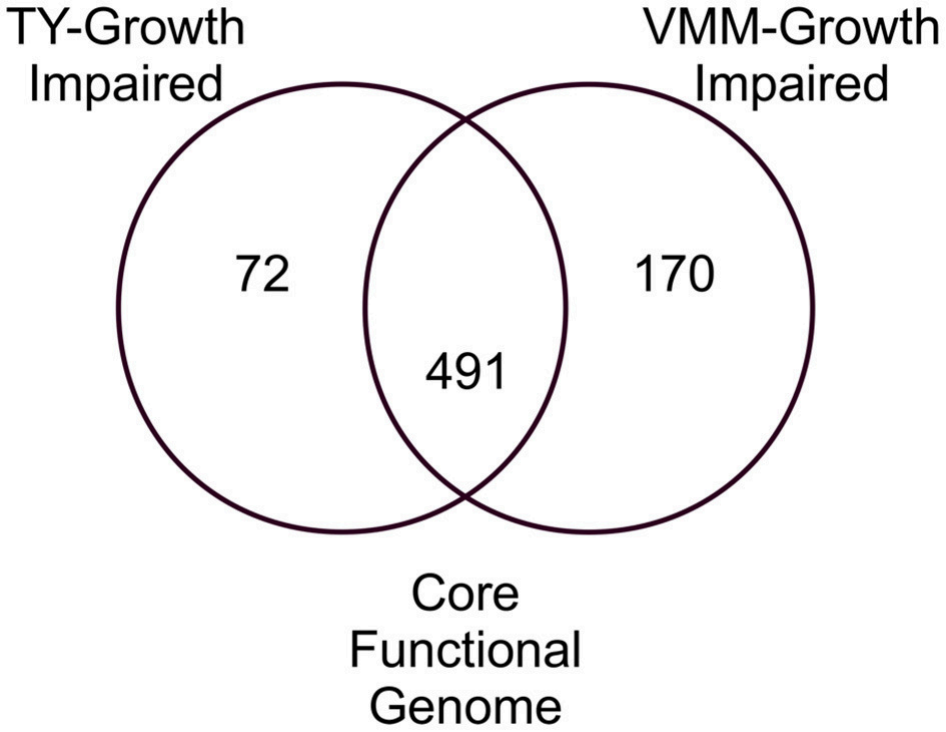
**Venn diagram of growth impaired genes observed for growth on TY and VMM-mannitol**. Growth impaired genes observed uniquely on TY or VMM were assigned to TY-growth impaired or VMM-growth impaired. Growth impaired genes observed in both treatments were assigned to the CFG. Genes without potential mariner insertion sites, or which had highly similar sequence redundancy in the RLV3841 genome were discounted.

**Figure 2:**
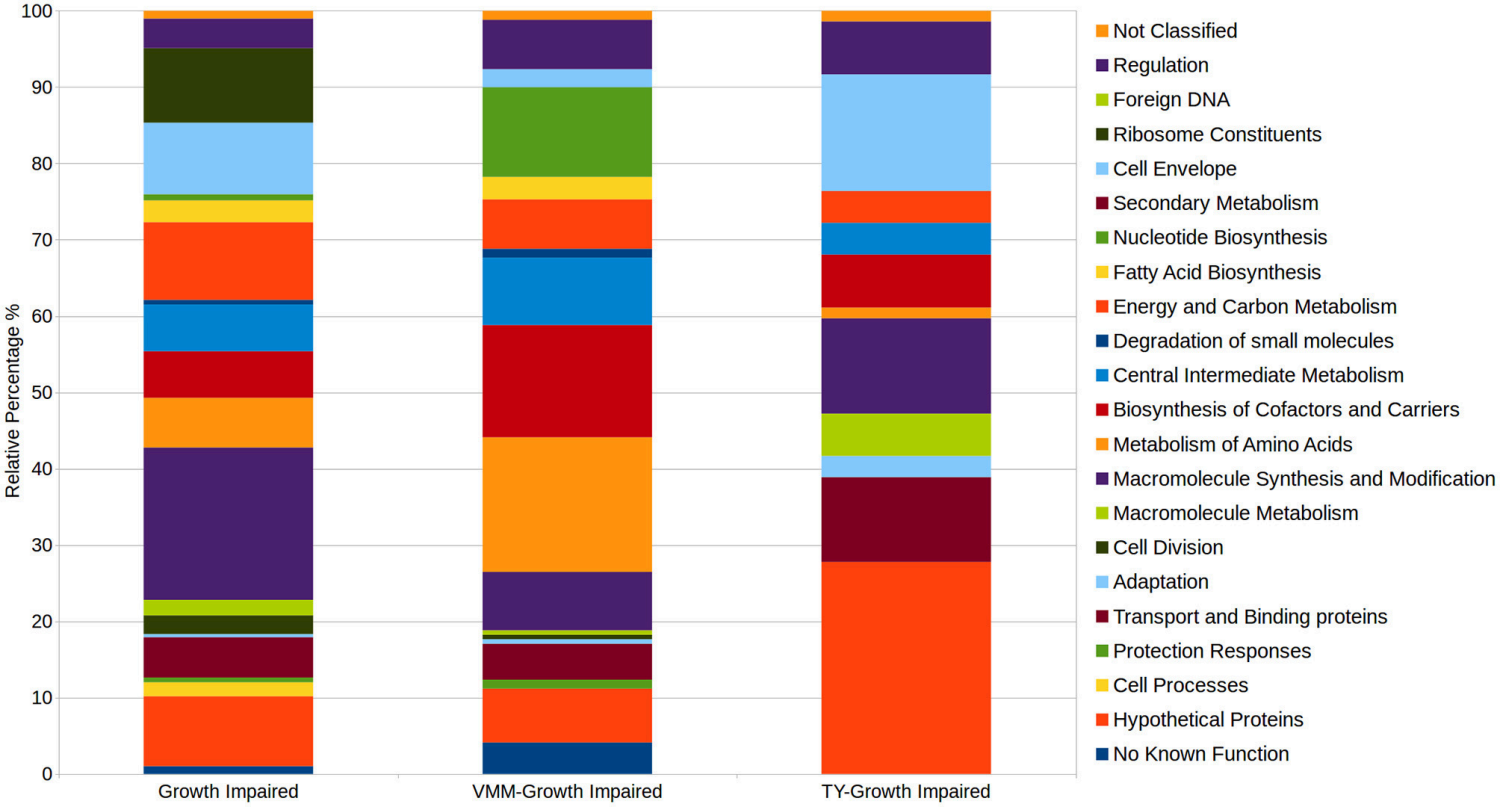
**Relative distributions of Riley functional gene classifications within growth impaired categories**. Growth impaired genes on both VMM-Mannitol and TY media were assigned to the CFG. CFG, TY-growth impaired, and VMM-mannitol growth impaired genes were assigned Riley functional classification based on Young et al. (2006). The relative abundance of genes within observed Riley functional groups were then calculated within each growth impaired category.

Comparison of the VMM-mannitol INSeq dataset with the TY INSeq dataset identified a set of genes that when mutated conferred a GI phenotype under both conditions; these 491 genes were assigned to the CFG (Figure 1). Whereas, 170 and 72 genes, when interrupted by Tn insertion, resulted in a condition dependent impaired ability to grow on VMM (VGI) and TY (TGI), respectively. Genes within the CFG were represented by 5 major Riley classification groups: macro-molecule synthesis and metabolism (20.0%), energy and carbon metabolism (10.2%), ribosomal constituents (9.8%), cell envelope (9.4%), and conserved hypothetical proteins (9.2%) (Figure 2). Genes that gave rise to a VGI phenotype were composed of 3 major functional groups: metabolism of amino acids (17.6%), biosynthesis of co-factors and carriers (14.7%), and nucleotide biosynthesis (11.8%). While the 4 major groups of TGI genes consisted of hypothetical proteins (27.8%), cell envelope (15.3%), macro-molecule synthesis and metabolism (12.5%), and transport and binding proteins (11.1%).

Open reading frames annotated as hypothetical proteins represent approximately 25.2% of the RLV3841 genome. Of these, 103 hypothetical proteins were observed to have a GI phenotype on VMM, TY, or both growth conditions (Table 1). Sequence duplication within the RLV3841 genome resulted in 20 annotated hypothetical proteins not being assayed for a growth phenotype using INSeq, due to multiple potential alignments of sequenced Tn insertion tags.

**Table 1:** **Growth impaired hypothetical proteins**.

		**VMM INSeq Data**				**TY INSeq Data**						
**Locus**	**Growth phenotype**	**N**	**Insertion density**	**Average read depth**	**Gene state**	**N**	**Insertion density**	**Average read depth**	**Gene state**	**Riley key**	**Annotation**	
**CORE FUNCTIONAL GENOME HYPOTHETICAL PROTEINS**												
RL1763	GI	2	0	0	ES	2	0	0	ES	0.0.0	Hypothetical protein	
RL2220	GI	7	0.57	29.75	ES	7	0.57	30.25	ES	0.0.0	Hypothetical protein	
RL2402	GI	13	0.46	13.5	GD	13	0.46	11.33	GD	0.0.0	Hypothetical protein	
RL2581	GI	6	0	0	ES	6	0.33	1.5	ES	0.0.0	Hypothetical protein	
RL4714	GI	1	0	0	ES	1	1	2	GD	0.0.0	Hypothetical protein	
RL0128	GI	8	0.13	1	ES	8	0	0	ES	0.0.1	Conserved hypothetical protein	
RL0393	GI	8	0.38	9.33	GD	8	0.5	4.25	GD	0.0.1	Conserved hypothetical protein	
RL0412	GI	10	0	0	ES	10	0	0	ES	0.0.1	Conserved hypothetical protein	
RL0548	GI	7	0	0	ES	7	0.14	2	ES	0.0.1	Conserved hypothetical protein	
RL0612	GI	2	0	0	ES	2	0	0	GD	0.0.1	Conserved hypothetical protein	
RL0620	GI	7	0	0	GD	7	0	0	GD	0.0.1	Conserved hypothetical protein	
RL1432	GI	5	0.2	7	ES	5	0.6	4.33	GD	0.0.1	Conserved hypothetical protein	
RL1566	GI	11	0.18	5	ES	11	0	0	ES	0.0.1	Conserved hypothetical protein	
RL2245	GI	8	0.13	22	ES	8	0.25	30	GD	0.0.1	Conserved hypothetical protein with RimI domain, putative N-acetyltransferase	
RL3754	GI	6	0.17	8	ES	6	0	0	ES	0.0.1	Conserved hypothetical protein	
RL3757	GI	5	0.6	16	GD	5	0.4	33.5	ES	0.0.1	Conserved hypothetical protein	
RL3762	GI	14	0	0	ES	14	0.07	1	ES	0.0.1	Conserved hypothetical exported protein	
RL4061	GI	10	0	0	ES	10	0.3	1.67	GD	0.0.1	Conserved hypothetical protein	
RL4324	GI	1	0	0	ES	1	0	0	GD	0.0.1	Conserved hypothetical exported protein	
RL4434	GI	3	0	0	ES	3	0	0	GD	0.0.1	Conserved hypothetical protein	
RL4437	GI	8	0	0	GD	8	0.13	2	GD	0.0.1	Conserved hypothetical protein	
RL4733	GI	8	0	0	ES	8	0	0	ES	0.0.1	Conserved hypothetical protein	
RL0126	GI	8	0	0	ES	8	0.13	3	ES	0.0.2	Conserved hypothetical protein	
RL0144	GI	5	0	0	ES	5	0	0	ES	0.0.2	Conserved hypothetical protein	
RL0456A	GI	4	0	0	NE	4	0	0	NE	0.0.2	Conserved hypothetical protein	
RL0619	GI	4	0.25	1	GD	4	0.25	1	GD	0.0.2	Conserved hypothetical protein	
RL0626	GI	13	0.46	12.67	GD	13	0.62	14.25	GD	0.0.2	Conserved hypothetical protein	
RL0936	GI	30	0.07	1	ES	30	0.17	2	GD	0.0.2	Conserved hypothetical protein (TPR repeat family)	
RL1094	GI	17	0.12	8	ES	17	0.18	5.33	ES	0.0.2	Conserved hypothetical protein	
RL1116	GI	9	0.11	18	ES	9	0.11	39	ES	0.0.2	Conserved hypothetical protein	
RL1569	GI	21	0.05	1	ES	21	0.14	1	ES	0.0.2	Conserved hypothetical protein	
RL1598	GI	6	0	0	ES	6	0.17	1	GD	0.0.2	Conserved hypothetical protein	
RL1706	GI	3	0	0	ES	3	0	0	GD	0.0.2	Conserved hypothetical protein in NADH-ubiquinone oxidoreductase region	
RL2034	GI	8	0.5	3.75	GD	8	0.38	1	ES	0.0.2	Conserved hypothetical protein	
RL2042	GI	29	0.28	8.5	ES	29	0.17	5.4	GD	0.0.2	Conserved hypothetical protein	
RL2056	GI	5	0.2	1	GD	5	0.2	2	GD	0.0.2	Conserved hypothetical protein	
RL2207	GI	6	0.17	1	ES	6	0.17	2	GD	0.0.2	Conserved hypothetical protein	
RL2232	GI	11	0.09	1	ES	11	0	0	ES	0.0.2	Conserved hypothetical protein	
RL2584	GI	9	0	0	ES	9	0.11	2	ES	0.0.2	Conserved hypothetical protein	
RL2697	GI	5	0.2	21	ES	5	0.2	22	ES	0.0.2	Conserved hypothetical exported protein	
RL3259	GI	1	0	0	NE	1	0	0	NE	0.0.2	Conserved hypothetical protein	
RL3314	GI	8	0	0	ES	8	0	0	ES	0.0.2	Conserved hypothetical protein	
RL3464	GI	15	0.27	1.5	GD	15	0.53	2.63	GD	0.0.2	Conserved hypothetical protein	
RL3948A	GI	31	0.06	2	ES	31	0.16	1	ES	0.0.2	Conserved hypothetical protein	
RL3967	GI	19	0	0	ES	19	0.53	3.5	GD	0.0.2	Conserved hypothetical protein	
RL4037	GI	8	0.5	3.25	GD	8	0.63	6.8	GD	0.0.2	Conserved hypothetical protein	
RL4280	GI	20	0.4	2.5	GD	20	0.25	4	ES	0.0.2	Conserved hypothetical protein	
RL4523	GI	13	0.23	7.67	ES	13	0.23	12.33	ES	0.0.2	Conserved hypothetical exported protein	
RL4562	GI	10	0.1	3	ES	10	0.1	4	ES	0.0.2	Conserved hypothetical exported protein	
RL4734	GI	14	0	0	ES	14	0	0	ES	0.0.2	Conserved hypothetical protein	
**VMM-GROWTH IMPAIRED HYPOTHETICAL PROTEINS**												
RL0044	VGI	13	0.62	22.88	ES	13	0.69	32.33	NE	0.0.0	Hypothetical protein	
RL1117	VGI	6	0.5	15.33	ES	6	0.67	8	NE	0.0.0	Hypothetical protein	
RL1628	VGI	39	0.64	8.32	ES	39	0.67	10.35	NE	0.0.0	Hypothetical protein	
RL1946	VGI	13	0.62	1.75	GD	13	0.69	3.33	NE	0.0.0	Hypothetical protein	
RL2117A	VGI	33	0.76	11.36	ES	33	0.79	13.31	NE	0.0.0	Hypothetical protein	
RL2154	VGI	16	0.69	7.55	GD	16	0.75	7.25	NE	0.0.0	Hypothetical protein	
RL2611	VGI	10	0.5	11.6	ES	10	0.9	14	NE	0.0.0	Hypothetical protein	
RL1524	VGI	5	0.6	3.33	GD	5	0.8	9.75	NE	0.0.1	Conserved hypothetical protein	
RL3760	VGI	7	0	0	ES	7	0.29	9	NE	0.0.1	Conserved hypothetical protein	
RL4043	VGI	2	0.5	2	ES	2	1	29	NE	0.0.1	Conserved hypothetical protein	
RL4066	VGI	24	0.42	2.8	GD	24	0.79	9.68	NE	0.0.1	Conserved hypothetical protein	
RL0571	VGI	3	0	0	ES	3	1	26.67	NE	0.0.2	Conserved hypothetical protein	
RL0960	VGI	10	0.3	1.33	GD	10	0.5	2.6	NE	0.0.2	Conserved hypothetical protein (Sua5 family)	
RL1523	VGI	4	0.25	2	GD	4	1	4.25	NE	0.0.2	Conserved hypothetical protein	
RL2210	VGI	5	0.2	1	GD	5	0.4	1	NE	0.0.2	Conserved hypothetical protein	
RL2289	VGI	3	0.33	1	GD	3	1	19	NE	0.0.2	Conserved hypothetical protein	
RL2291	VGI	7	0.71	2	GD	7	0.86	20	NE	0.0.2	Conserved hypothetical protein	
RL2527	VGI	13	0.31	8.5	GD	13	0.69	6.78	NE	0.0.2	Conserved hypothetical exported protein	
RL4728	VGI	13	0.23	6	ES	13	0.69	6.11	NE	0.0.2	Conserved hypothetical protein	
**TY-GROWTH IMPAIRED HYPOTHETICAL PROTEINS**												
RL1527	TGI	2	1	24	NE	2	1	3.5	GD	0.0.1	Conserved hypothetical protein	
RL1562	TGI	9	0.56	14.8	NE	9	0.56	16.4	ES	0.0.1	Conserved hypothetical protein	
RL1618A	TGI	17	1	9.82	NE	17	0.65	2.18	GD	0.0.1	Conserved hypothetical protein	
RL2307	TGI	4	0.75	5.67	NE	4	0.75	10	GD	0.0.1	Conserved hypothetical protein	
RL4065	TGI	3	0.67	1	NE	3	0	0	NE	0.0.1	Conserved hypothetical protein	
RL4716	TGI	18	1	37.17	NE	18	0.61	6.55	GD	0.0.1	Conserved hypothetical exported protein	
RL0109	TGI	8	0.75	8.5	NE	8	0.25	2.5	GD	0.0.2	Conserved hypothetical protein	
RL0890	TGI	16	0.81	19.54	NE	16	0.5	13.75	ES	0.0.2	Conserved hypothetical protein	
RL1526	TGI	11	0.91	34.9	NE	11	0.64	5.43	GD	0.0.2	Conserved hypothetical protein	
RL1528	TGI	11	0.91	14.3	NE	11	0.45	7.2	GD	0.0.2	Conserved hypothetical protein	
RL2086	TGI	6	0.83	5.8	NE	6	0.5	1.33	GD	0.0.2	Conserved hypothetical exported protein	
RL2142	TGI	17	0.71	13.33	NE	17	0.65	17.55	ES	0.0.2	Conserved hypothetical protein	
RL2542	TGI	2	1	4.5	NE	2	0	0	GD	0.0.2	Conserved hypothetical protein	
RL2625	TGI	15	0.8	9.08	NE	15	0.47	3.71	GD	0.0.2	Conserved hypothetical protein	
RL2641	TGI	9	0.67	14	NE	9	0.56	5.8	GD	0.0.2	Conserved hypothetical protein	
RL3499	TGI	14	0.93	17.08	NE	14	0.5	2.29	GD	0.0.2	Conserved hypothetical protein	
RL3500	TGI	18	1	34.89	NE	18	0.67	5.75	GD	0.0.2	Conserved hypothetical protein	
RL3761	TGI	14	0.93	13.23	NE	14	0.21	1	ES	0.0.2	Conserved hypothetical exported protein	
RL4016	TGI	5	0.8	27.5	NE	5	0	0	GD	0.0.2	Conserved hypothetical protein	
RL4503	TGI	4	0.75	3	NE	4	1	4.75	GD	0.0.2	Conserved hypothetical protein	
**PLASMID GROWTH IMPAIRED HYPOTHETICAL PROTEINS**												
pRL100111	PGI	38	0.45	3.18	GD	38	0.45	3.71	ES	0.0.0	Hypothetical protein	
pRL70135	PGI	17	0.47	22.75	ES	17	0.65	23	ES	0.0.0	Hypothetical protein	
pRL120721	PGI	6	0.17	1	ES	6	0.33	2.5	ES	0.0.1	Conserved hypothetical protein	
pRL100012	PGI	3	0	0	ES	3	0	0	ES	0.0.2	Conserved hypothetical protein	
pRL110465	PGI	8	0.13	1	GD	8	0.13	2	GD	0.0.2	Conserved hypothetical protein	
pRL70167	PGI	11	0.27	11	ES	11	0.27	19	ES	0.0.2	Conserved hypothetical protein	
pRL80098	PGI	11	0.18	9.5	ES	11	0.64	8.14	GD	0.0.2	Conserved hypothetical protein	
**PLASMID/(VMM/TY)-GROWTH IMPAIRED HYPOTHETICAL PROTEINS**												
pRL100010	PVGI	3	0.33	3	GD	3	1	11.67	NE	0.0.2	Conserved hypothetical protein	
pRL100149	PVGI	58	0.83	14.56	ES	58	0.83	18.54	NE	0.0.2	Conserved hypothetical protein	
pRL110044	PVGI	6	0.33	1.5	GD	6	0.83	4	NE	0.0.2	Conserved hypothetical protein	
pRL110108	PVGI	17	0.76	9.85	ES	17	0.76	13.38	NE	0.0.2	Conserved hypothetical protein	
pRL110351A	PTGI	10	0.7	9.71	NE	10	0.6	14.67	ES	0.0.2	Conserved hypothetical protein	
pRL70166	PVGI	9	0.33	6.33	ES	9	0.67	6.83	NE	0.0.2	Conserved hypothetical protein	
pRL90280	PVGI	6	0.5	4.33	ES	6	0.83	6.6	NE	0.0.2	Conserved hypothetical protein	
**Locus**	**Growth phenotype**	**N**	**Insertion density**	**Average read depth**	**Gene state**	**N**	**Insertion density**	**Average read depth**	**Gene state**	**Riley key**	**Annotation**	**Sequence duplications**
**GROWTH IMPAIRED OR PLASMID GROWTH IMPAIRED HYPOTHETICAL PROTEINS WITH SEQUENCE DUPLICATION**												
pRL70102	PGI	4	0	0	ES	4	0	0	ES	0.0.0	Hypothetical protein	pRL70102, pRL110582, downstream pRL100152, upstream pRL100166
RL1481	GI	6	0	0	ES	6	0	0	ES	0.0.0	Hypothetical protein	RL1481, 3′-end of RL00456B, 5′-end of RL0457, 3′-end of pRL100088, 5′-end of pRL100089
pRL110342	PGI	2	0	0	ES	2	0	0	ES	0.0.0	Hypothetical protein	upstream pRL100046, upstream RL2679
pRL100089	PGN	5	0.4	5.5	NE	5	0.6	5.33	NE	0.0.1	Conserved hypothetical protein	pRL100089, RL1481
pRL70033	PGI	8	0	0	ES	8	0	0	ES	0.0.1	Conserved hypothetical protein	pRL70033, pRL70047D, pRL70147
pRL70047	PGI	6	0	0	ES	6	0	0	ES	0.0.1	Conserved hypothetical protein	pRL70142, pRL70047
pRL70142	PGI	6	0	0	ES	6	0	0	ES	0.0.1	Conserved hypothetical protein	pRL70142, pRL70047
pRL70179	PGI	3	0	0	ES	3	0	0	ES	0.0.1	Conserved hypothetical protein	pRL70169, pRL70179, PRL100095
pRL120379	PGI	35	0	0	ES	35	0	0	ES	0.0.1	Conserved hypothetical protein	pRL90004, pRL90319, pRL100469, pRL120379
pRL90004	PGI	35	0	0	ES	35	0	0	ES	0.0.1	Conserved hypothetical protein	pRL90004, pRL90319, pRL100469, pRL120379
pRL120650	PGI	6	0	0	ES	6	0	0	ES	0.0.1	Conserved hypothetical protein	RL2139, RL0835, pRL120650
RL0835	GI	7	0	0	ES	7	0.14	21	ES	0.0.1	Conserved hypothetical protein	RL2139, RL0835, pRL120650
RL2139	GI	6	0	0	ES	6	0	0	ES	0.0.1	Conserved hypothetical protein	RL2139, RL0835, pRL120650
pRL110353	PGN	4	0.25	2	NE	4	0.25	3	NE	0.0.2	Conserved hypothetical protein	pRL110353, RL2153
RL2153	GI	4	0.25	3	GD	4	0.25	4	GD	0.0.2	Conserved hypothetical protein	pRL110353, RL2153
pRL100095	PGI	2	0	0	NE	2	0	0	NE	0.0.2	Conserved hypothetical protein	pRL70169, pRL70179, PRL100095
pRL70169	PGI	2	0	0	ES	2	0	0	ES	0.0.2	Conserved hypothetical protein	pRL70169, pRL70179, PRL100095
pRL100469	PGI	35	0	0	ES	35	0	0	ES	0.0.2	Conserved hypothetical protein	pRL90004, pRL90319, pRL100469, pRL120379
pRL90319	PGI	35	0	0	ES	35	0	0	ES	0.0.2	Conserved hypothetical protein, possible fusion protein	pRL90004, pRL90319, pRL100469, pRL120379

### Growth curve analysis of two predicted VMM-growth impaired mutants

**Figure 3:**
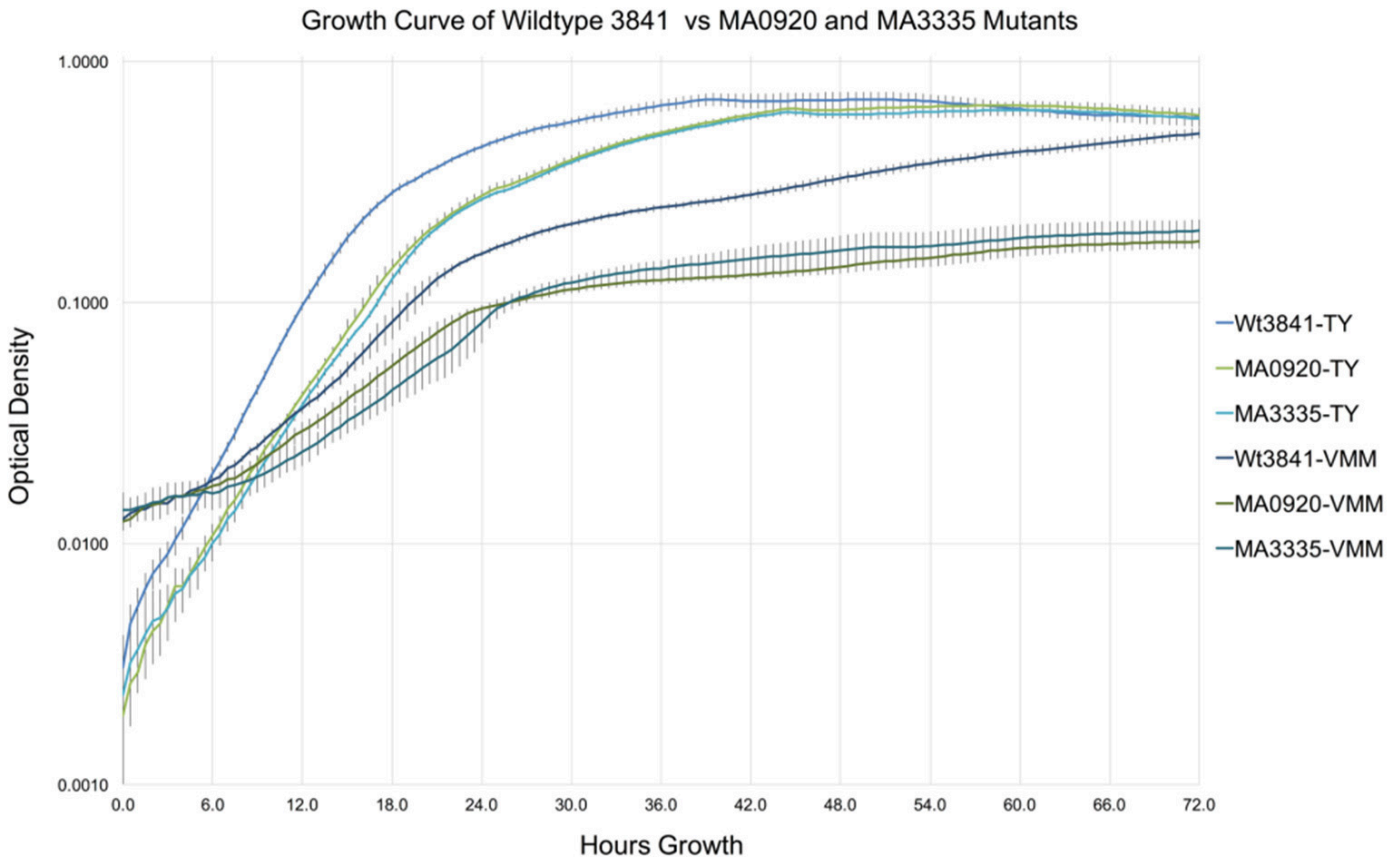
**Growth curve comparison of RLV3841, MA0920, and MA3335 in TY and VMM**. Growth curves represent the mean OD_600_ of 7 replicates measured at 30 min intervals over 72 h in TY and VMM-Mannitol liquid growth media; error bars indicate the standard deviation of the mean. MA0920 and MA3335 had longer mean generation times than the wildtype in TY and VMM-mannitol. After 72 h of growth in VMM-mannitol, MA0920, and MA3335 reached cell densities that were ~1/3 of the wildtype.

Growth curves of RLV3841, MA0920 (INSeq predicted RL0920 VGI), and MA3335 (INSeq predicted RL3335 VGI), in TY and VMM-Mannitol broth over 72 h are shown in Figure 3. After 72 h growth RLV3841, MA0920, and MA3335 reached final mean OD_600_ readings of 0.696, 0.657, and 0.628 on TY; and 0.588, 0.179, and 0.198 on VMM, respectively. Mean generation times for RLV3841, MA0920, and MA3335 on TY were 2.5, 3.0, and 3.0 h in early exponential phase; and 3.5, 4.5, and 4.0 h in late exponential phase. The MGTs in VMM-Mannitol were observed to be 6.0, 7.5, and 8.5 h in early exponential phase for RLV3841, MA0920, and MA3335 respectively. In late exponential phase, MA0920 and MA3335 halted growth and did not complete an additional doubling, whereas RLV3841 wildtype continued to double with a MGT of 9.5 h.

### The genetics of central carbon metabolism for growth on mannitol

**Figure 4:**
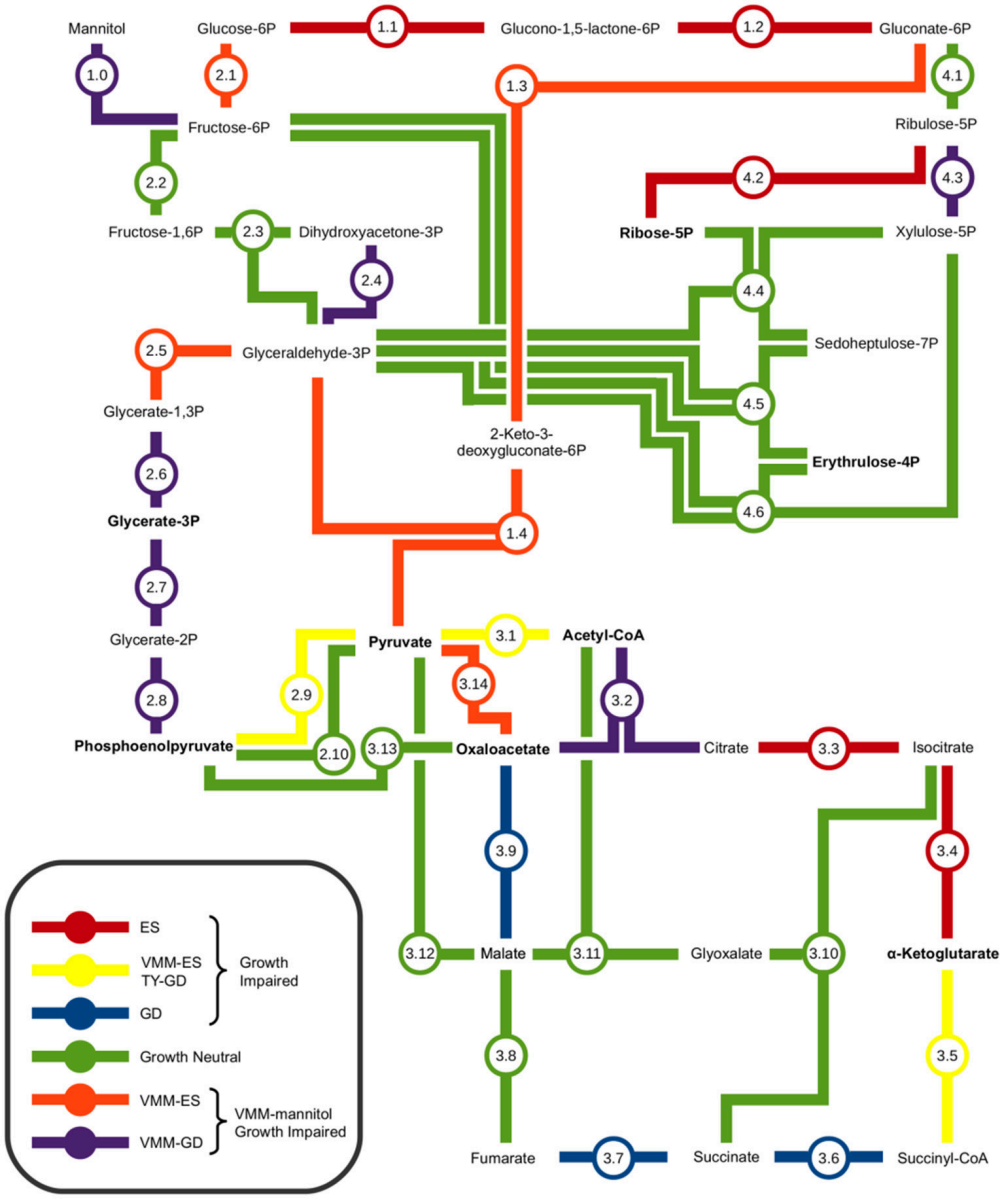
**Metabolic map of central carbon metabolism of ***Rhizobium leguminosarum*** bv. ***viciae*** 3841**. Interconnections between the Embden-Meyerhof-Parnas pathway, Entner-Doudoroff pathway, Pentose Phosphate pathway, and Tri-Carboxylic Acid cycle were based on the proposed central carbon metabolism of *Sinorhizobium meliloti* and *Agrobacterium tumefaciens* (Fuhrer et al., 2005; Geddes and Oresnik, 2014). Amino acid precursors are indicated in bold text. The impact of mutations in each metabolic step on growth were determined by contrasting results from TY and VMM-mannitol INSeq experiments. Genes observed to be growth defective or essential exclusively on VMM-Mannitol were concluded to be involved in central carbon metabolism of mannitol. Genes observed to be GD or ES on TY and VMM-mannitol were grouped, and assumed to have roles in central carbon metabolism that were not mannitol dependent.

INSeq was used to identify a potential minimal central carbon metabolism pathway for growth on mannitol. Figure 4, and accompanying Table 2, provide a metabolic map illustrating the interconnections of the Embden-Meyerhof-Parnas (EMP) pathway, Entner-Doudoroff (ED) pathway, pentose phosphate (PP) pathway, and tricarboxylic acid cycle (TCA) that compose central carbon metabolism, with an overlay of the observed growth impaired phenotypes. It was observed that the genes required for mannitol uptake and conversion to fructose-6P (F6P) were VGI. Genes required for conversion of F6P to pyruvate were observed to be VGI within the ED pathway. Genes required for conversion of F6P into glyceraldehyde-3P (GA3P), via the upper EMP pathway, were observed to have no impact on growth when mutated. Genes required for the conversion of GA3P to pyruvate as part of the lower EMP pathway were found to be VGI when mutated. Assimilation of pyruvate into the TCA cycle was observed to be VGI via more than one metabolic pathway. Mutation of genes within the TCA cycle were observed to result in GI or VGI phenotypes, with the exception of a growth neutral step at the conversion between fumarate and malate (Figure 3; Table 2).

**Table 2:** **Growth phenotypes of genes involved in RLV3841 central carbon metabolism**.

					**VMM INSeq**			**TY INSeq**				
**Metabolic step**	**RLV locus**	**RLV symbol**	**Putative product**	**Growth phenotype**	**Insertion density**	**Average read depth**	**Gene state**	**Insertion density**	**Average read depth**	**Gene state**	**Riley key**	**Riley functional information**
**MANNITOL UPTAKE AND CONVERSION TO FRUCTOSE**												
1.0	RL4219	–	Transcriptional regulator	GN	0.86	16.67	NE	0.79	25.27	NE	6.3.10	Putative deor family transcriptional regulator (repressor) of sorbitol/mannitol operon
1.0	RL4215	–	Mannitol ABC transporter ATP-binding protein	VGI	0.41	1.71	GD	0.88	12.6	NE	1.5.3	Putative ATP-binding component of ABC transporter CUT1 mannitol transporter (S. Mel SBP homolog smc01496 induced by dulcitol, sorbitol, mannitol)
1.0	RL4216	*mtlG*	Mannitol transmembrane permease component of ABC transporter	VGI	0.56	3.2	GD	1	34.11	NE	1.5.3	Putative permease component of ABC transporter CUT1 mannitol transporter (S. Mel SBP homolog smc01496 induced by dulcitol, sorbitol, mannitol)
1.0	RL4217	*mtlF*	Mannitol transmembrane permease component of ABC transporter	VGI	0.53	2.78	GD	0.94	36.06	NE	1.5.3	Putative permease component of ABC transporter CUT1 mannitol transporter (S. Mel SBP homolog smc01496 induced by dulcitol, sorbitol, mannitol)
1.0	RL4218	*mtlE*	Mannitol-binding component of ABC transporter	VGI	0.48	2.3	GD	0.9	26.11	NE	1.5.3	Putative SBP of ABC transporter CUT1 mannitol transporter (S. Mel SBP homolog smc01496 induced by dulcitol, sorbitol, mannitol)
1.0	RL4214	*mtlK*	Mannitol 2-dehydrogenase	VGI	0.12	7.5	GD	0.88	24.8	NE	3.4.3	Putative mannitol 2-dehydrogenase
1.0	RL0098	–	Mannitol dehydrogenase	GN	0.93	17	NE	0.93	17.62	NE	3.4.3	Putative mannitol dehydrogenase
1.0	RL0502	*frk*	Fructokinase	VGI	0.21	3	GD	1	15.71	NE	3.5.5	Fructokinase
**ENTNER–DOUDOROFF PATHWAY**												
1.1	RL0753	*zwf1*	Putative glucose-6-phosphate 1-dehydrogenase	GI	0.05	1	ES	0.09	1	ES	3.5.6	Putative glucose-6-phosphate 1-dehydrogenase
1.1	RL1315	*zwf1*	Glucose-6-phosphate 1-dehydrogenase	GN	0.93	18.72	NE	1	18.7	NE	3.5.6	Putative glucose-6-phosphate 1-dehydrogenase
1.1	pRL120561	–	Glucose-6-phosphate 1-dehydrogenase	PGN	1	19	NE	1	14.5	NE	6.3.0	Putative XRE family transcriptional regulator
1.2	RL0752	*pgl*	6-phosphogluconolactonase	GI	0	0	ES	0	0	ES	3.5.6	Putative 6-phosphogluconolactonase
1.3	RL0751	*edd*	Phosphogluconate dehydratase	VGI	0	0	ES	1	8.73	NE	3.3.3	Putative phosphogluconate dehydratase
1.4	RL4162	*eda*	Keto-hydroxyglutarate-aldolase/keto-deoxy-phosphogluconate aldolase	VGI	0.13	14	ES	0.88	3.14	NE	3.3.3	Putative 2-dehydro-3-deoxyphosphogluconate aldolase
**EMBDEN-MEYERHOF-PARNAS PATHWAY**												
2.1	RL0504	*pgi*	Glucose-6-phosphate isomerase	VGI	0.06	30	ES	0.76	10.69	NE	3.5.5	Putative glucose-6-phosphate isomerase
2.2	RL3322	*pfp*	Pyrophosphate-fructose-6-phosphate 1-phosphotransferase	GN	1	16.8	NE	0.95	21.79	NE	3.5.5	Putative pyrophosphate–fructose 6-phosphate 1-phosphotransferase
2.3	RL4012		Fructose-bisphosphate aldolase	GN	0.86	22.42	NE	0.93	21.23	NE	3.5.5	Putative fructose-bisphosphate aldolase
2.3	pRL120027		Aldolase	PGN	0.86	23.92	NE	0.93	21.46	NE	3.3.15	Putative aldolase
2.3	pRL120196		Fructose-1,6-bisphosphate aldolase	PGN	0.95	20.67	NE	1	22.53	NE	3.3.9	Putative fructose-bisphosphate aldolase
2.4	RL2513	*tpiA*	Triosephosphate isomerase	GN	0.9	14.56	NE	0.9	24.22	NE	3.6.0	Putative triosephosphate isomerase
2.4	pRL120209	*tpiA*	Triosephosphate isomerase	PVGI	0.38	2.33	GD	0.88	8.14	NE	3.6.0	Putative triosephosphate isomerase
2.5	RL4007	*gap*	Glyceraldehyde-3-phosphate dehydrogenase	VGI	0	0	ES	1	9	NE	3.5.1	Putative glyceraldehyde-3-phosphate dehydrogenase
2.6	RL4011	*pgk*	Phosphoglycerate kinase	VGI	0.25	3.67	GD	0.83	38.2	NE	3.5.1	Putative phosphoglycerate kinase
2.7	RL0179	*gpmA*	Phosphoglyceromutase	VGI	0.36	1.5	GD	0.73	9.88	NE	3.5.5	Putative 2,3-bisphosphoglycerate-dependent phosphoglycerate mutase (Phosphoglyceromutase) (PGAM) (BPG-dependent PGAM) (dPGM)
2.7	RL2655		Hypothetical protein	GN	0.88	10.71	NE	1	9.38	NE	0.0.0	Hypothetical protein
2.7	RL0954	*gpmB*	Phosphoglycerate mutase	GN	1	19.22	NE	0.89	21.38	NE	3.5.5	Putative phosphoglycerate mutase
2.7	RL1010	*gpmB*	Phosphoglycerate mutase	GN	0.83	15.2	NE	0.83	26.2	NE	3.5.5	Putative phosphoglycerate mutase
2.7	RL2997		Phosphoglycerate mutase	GN	1	34.29	NE	1	39.12	NE	3.5.5	Putative phosphoglycerate mutase
2.8	RL2239	*eno*	Phosphopyruvate hydratase	VGI	0.19	1	GD	0.81	4.77	NE	3.5.5	Putative enolase
2.9	RL4060	*pykA*	Pyruvate kinase	GI	0.23	8.67	ES	0.92	4.83	GD	3.5.5	Putative pyruvate kinase
2.10	RL1086	*ppdK*	Pyruvate phosphate dikinase	GN	0.84	21.26	NE	0.91	24.69	NE	3.5.5	Pyruvate, phosphate dikinase (pyruvate, orthophosphate dikinase)
**TRICARBOXYLIC ACID CYCLE**												
3.1	RL2241	*pdhA*	Pyruvate dehydrogenase subunit	GI	0	0	ES	0.18	1	GD	3.5.5	Putative pyruvate dehydrogenase subunit A
3.1	RL2242	*pdhB*	Pyruvate dehydrogenase subunit beta	GI	0	0	ES	0.22	1.25	GD	3.5.5	Putative pyruvate dehydrogenase subunit B
3.1	RL2243	*pdhC*	Dihydrolipoamide acetyltransferase component of pyruvate dehydrogenase complex	GI	0	0	ES	0	0	GD	3.5.5	Putative dihydrolipoamide acetyltransferase component of pyruvate dehydrogenase complex (PDC)
3.1	pRL80081	–	Hydrolase	PGN	0.86	9.33	NE	0.86	9.33	NE	3.3.15	Putative hydrolase
3.2	RL2234	*gltA*	Type II citrate synthase	VGI	0.7	4.32	GD	0.85	14.09	NE	3.5.8	Putative citrate synthase
3.2	RL2508	*gltA*	Citrate synthase II	GN	0.85	14.82	NE	1	13.54	NE	3.5.8	Putative citrate synthase II
3.2	RL2509	*citA*	Citrate synthase 2	GN	0.8	19	NE	0.8	19.17	NE	3.5.8	Putative citrate synthase I
3.3	RL4536	*acnA*	Aconitate hydratase	GI	0.05	1	ES	0.05	1	ES	3.5.8	Putative aconitate hydratase
3.4	RL2631	*icd*	Isocitrate dehydrogenase	GI	0.12	10.5	ES	0.12	8	ES	3.5.8	Putative isocitrate dehydrogenase [NADP]
3.5	RL4435	*sucA*	2-oxoglutarate dehydrogenase E1 component	GI	0.05	1.5	ES	0.23	1.5	GD	3.5.8	Putative 2-oxoglutarate dehydrogenase E1 component
3.5	RL4433	*citM*	Dihydrolipoamide succinyltransferase	GI	0.2	9	ES	0.3	4.33	GD	3.5.8	Putative dihydrolipoyllysine-residue succinyltransferase component of 2-oxoglutarate dehydrogenase
3.6	RL4436	*sucD*	Succinyl-CoA synthetase subunit alpha	GI	0.15	1	GD	0.23	1.33	GD	3.5.8	Putative succinyl-coa synthetase alpha chain
3.6	RL4438	*sucC*	Succinyl-CoA synthetase subunit beta	GI	0.09	1	GD	0.36	1	GD	3.5.8	Putative succinyl-coa synthetase beta chain
3.7	RL4443	*sdhB*	Succinate dehydrogenase iron-sulfur subunit	GI	0.13	3	GD	0.73	2.82	GD	3.5.8	Putative succinate dehydrogenase iron-sulfur protein
3.7	RL4444	*sdhA*	Succinate dehydrogenase flavoprotein subunit	GI	0.53	1.5	GD	0.76	3.77	GD	3.5.8	Putative succinate dehydrogenase flavoprotein subunit
3.7	RL4445	*sdhD*	Succinate dehydrogenase hydrophobic membrane anchor protein	GI	0.67	1.25	GD	0.67	4	GD	3.5.8	Putative succinate dehydrogenase hydrophobic membrane anchor protein
3.7	RL4446	*sdhC*	Succinate dehydrogenase cytochrome b556 subunit	GI	0.46	1.83	GD	0.77	4.6	GD	3.5.8	Putative succinate dehydrogenase cytochrome b556 subunit
3.8	RL2701	*fumC*	Fumarate hydratase class II	GN	0.94	18.56	NE	0.88	19.8	NE	3.5.8	Putative fumarate hydratase class II
3.8	RL2703	*fumA*	Fumarate hydratase class I	GN	0.93	23.36	NE	0.96	20.81	NE	3.5.8	Putative fumarate hydratase class I, aerobic
3.9	RL4439	*mdh*	Malate dehydrogenase	GI	0.25	1.33	GD	0.17	1	GD	3.5.8	Putative malate dehydrogenase
3.10	RL0761	-	Isocitrate lyase	GN	0.91	17.29	NE	0.83	25.79	NE	3.3.5	Putative isocitrate lyase
3.11	RL0054	*glcB*	Malate synthase G	GN	0.88	20	NE	1	24.06	NE	3.3.5	Putative malate synthase
3.12	RL0407	*maeB*	Malic enzyme	GN	0.86	18.97	NE	0.89	24.74	NE	3.5.8	Putative NADP-dependent malate dehydrogenase
3.12	RL2671	*maeB*	Malic enzyme	GN	0.83	31.47	NE	0.96	26.41	NE	3.5.8	Putative NADP-dependent malic enzyme
3.13	RL0037	*pckA*	Phosphoenolpyruvate carboxykinase	GN	1	16.09	NE	0.91	21.55	NE	3.3.4	Putative phosphoenolpyruvate carboxykinase
3.14	RL4638	–	Pyruvate carboxylase	VGI	0	0	ES	0.94	29.35	NE	3.5.8	Putative pyruvate carboxylase
**PENTOSE PHOSPHATE PATHWAY**												
4.1	RL2807	*gnd*	6-phosphogluconate dehydrogenase	GN	0.92	10.27	NE	0.92	12.27	NE	3.5.6	Putative phosphogluconate dehydrogenase
4.1	RL3998	*gntZ*	6-phosphogluconate dehydrogenase	GN	0.89	20.68	NE	1	21.89	NE	3.5.6	Putative 6-phosphogluconate dehydrogenase,decarboxylating
4.2	RL2547	*rpiB*	Ribose-5-phosphate isomerase B	GN	0.57	25.75	NE	0.71	16	NE	3.3.9	Putative ribose-5-phosphate isomerase B
4.2	RL2698	*rpiA*	Ribose-5-phosphate isomerase A	GI	0	0	ES	0.33	7	ES	3.3.9	Putative ribose-5-phosphate isomerase A
4.2	pRL120210	–	Ribose-5-phosphate isomerase B	PVGI	0.83	5.2	GD	0.83	21.8	NE	3.3.9	Putative ribose-5-phosphate isomerase B
4.3	RL2598	*rpe*	Ribulose-phosphate 3-epimerase	VGI	0.38	2.33	GD	0.63	9.2	NE	3.3.9	Putative ribulose phosphate 3-epimerase
4.3	pRL120033	–	D-allulose-6-phosphate 3-epimerase	PGN	1	20.6	NE	1	21	NE	3.3.9	Putative D-allulose-6-phosphate 3-epimerase
4.4, 4.6	RL2718	–	Transketolase	GN	1	16.23	NE	0.92	14.83	NE	3.3.15	Putative transketolase, alpha subunit, terpenoid biosynth?
4.4, 4.6	RL2719	–	Transketolase	GN	0.93	17.31	NE	0.93	24.38	NE	3.3.15	Putative transketolase, beta subunit, terpenoid biosynth?
4.4, 4.6	RL4006	*cbbT*	Transketolase	GN	0.67	8.38	NE	0.92	18.95	NE	3.5.1	Putative transketolase
4.4, 4.6	pRL100453	–	Transketolase	PGN	0.86	17.16	NE	0.9	22.46	NE	3.5.6	Putative transketolase
4.5	RL4203	*talB*	Transaldolase B	GN	0.56	4.2	NE	0.89	32.13	NE	3.3.9	Putative transaldolase B

### Plasmid growth impaired genes

**Figure 5:**
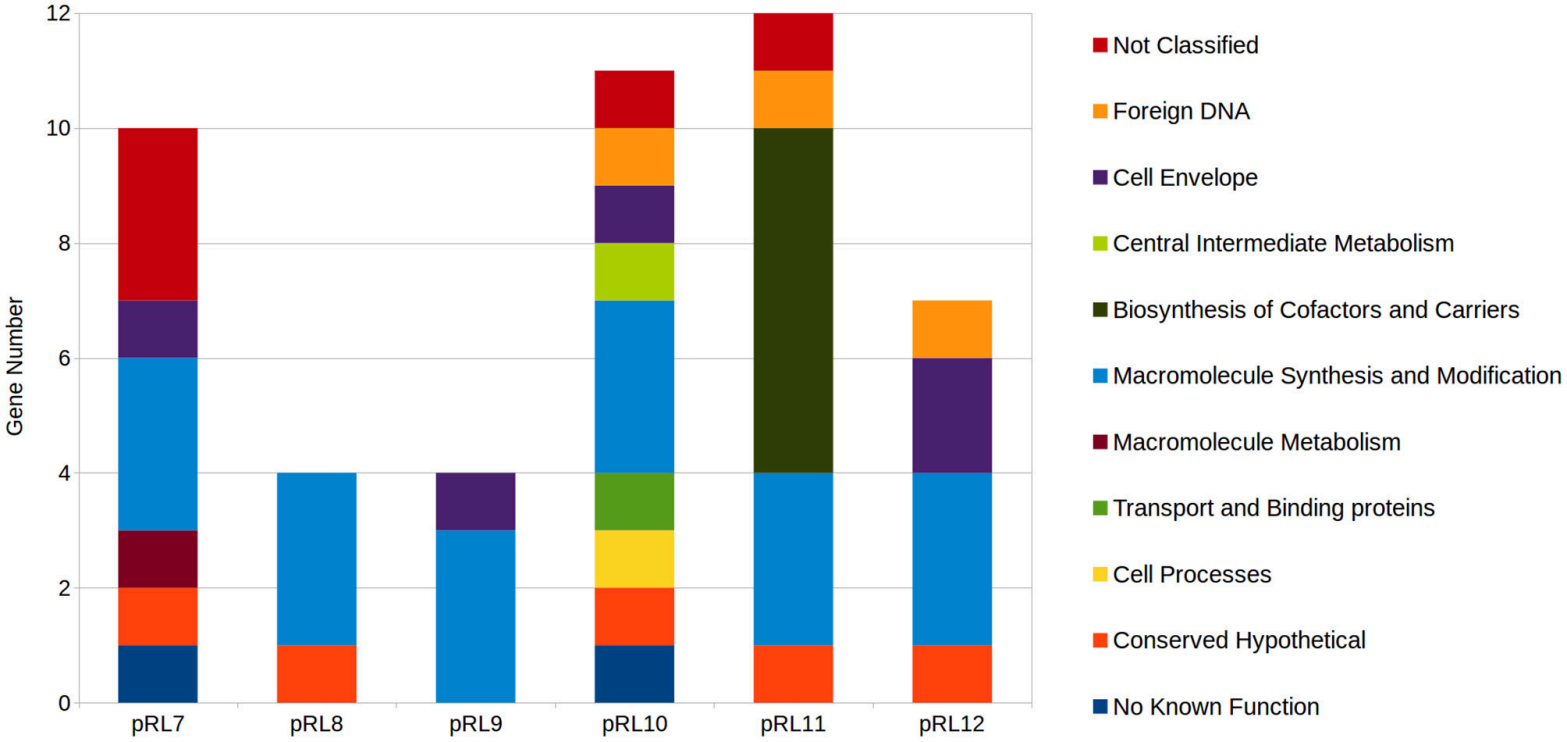
**Distribution of plasmid growth impaired genes by Riley functional classification across RLV3841 megaplasmids**. Plasmid borne genes observed to be growth defective or essential for growth on both TY and VMM-Mannitol were grouped. The contribution of growth defective or essential genes to megaplasmid stability, or growth of RLV3841 at an organism level, cannot be discriminated by INSeq analysis alone; therefore, the grouped growth defective and essential genes were assigned to plasmid growth impaired. Riley functional classifications were assigned to PGI genes for comparison of PGI profiles between megaplasmids.

Mutation of genes encoded on RLV3841 megaplasmids that resulted in a growth impaired phenotype were assigned as plasmid growth impaired (PGI), while plasmid genes that when mutated impaired growth on VMM or TY uniquely were designated PVGI and PTGI respectively. Collectively, 48 genes distributed across the 6 megaplasmids were predicted to result in a PGI phenotype when mutated (Supplementary File 1). The 48 PGI genes included 11 Riley functional classes (Figure 5). All 6 plasmids were observed to have a set of 3 replication protein encoding genes categorized as PGI. Plasmid pRL11 was observed to carry the most PGI biosynthetic genes including 6 genes of an 8 gene cluster predicted to code for cobalamin biosynthesis (Figure 5).

## Discussion

### The CFG of RLV3841

Young et al. (2006) used phylogeny of conserved genes and GC% to describe a core and accessory genome within the RLV3841. The present study helps to improve the level of resolution for distinguishing between the RLV3841 core genes and accessory genes using functional genetic screening. We propose that RLV3841 has a CFG which can be defined as the core set of genes required for normal growth, independent of any specific environmental condition. In this study we approximate the CFG of RLV3841 by contrasting INSeq generated data sets from growth on complex peptide rich and minimally defined media with mannitol as the sole carbon source. Cross referencing VGI and TGI chromosomal genes identified an overlapping set of 491 genes that we putatively assigned to the CFG of RLV3841, as their loss of function resulted in a GI phenotype that appears to be independent of the growth media used. The number of CFG genes was less than that of both the TGI and VGI genes, which represented 563 and 661 genes respectively (Figure 1). This is to be expected if the CFG represents a central set of genes required for core cellular functions.

Defining a CFG in RLV3841 provides context for subsequent INSeq and classical genetic studies. For example, the described CFG for RLV3841 will help explain if a mutation resulting in a GI phenotype in a plant associated environment is the result of impairing some aspect of the RLV3841 CFG or is instead the result of a plant specific interaction. This is particularly important for genes encoding hypothetical proteins of unknown function. Summarizing the distribution of gene functions in the CFG identified 20 functional groupings (Figure 2). Five major categories accounted for over half of the total CFG (287 genes). These 5 functional categories included: macromolecule synthesis and modification (98 genes), energy and carbon metabolism (50 genes), ribosome constituents (48 genes), cell envelope (46 genes), and hypothetical proteins (50 genes) (Figure 2). Hypothetical proteins aside, these 4 categories logically compose the majority of the CFG as they represent the central genes required for the synthesis of the major cellular components, central conversion of carbon for generation of reductant and ATP, production and modification of protein synthesis machinery, and synthesis of the cell envelope.

The fact that genes encoding hypothetical proteins was one of the five major categories assigned to the CFG reinforces the broadly acknowledged observation among geneticists that the function of many genes involved in core cellular processes still remain uncharacterized. INSeq is a powerful technique that can help identify hypothetical proteins required for survival in specific growth conditions, and ultimately will advance our rate of discovery in this large and under studied category of genes. For example, in this study we identified a total of 103 hypothetical proteins observed to have an impaired growth phenotype under at least one growth condition; of which 19 were VGI, 20 were TGI, 14 were plasmid associated, and the remainder belonged to the CFG (Table 1).

### Chromosomal VMM and TY specific growth impairment

Growth on minimal media requires the biosynthesis of several metabolites that can be scavenged from a complex growth media. Therefore, it was expected that there were more VGI genes compared to TGI genes, and that a substantial portion of the VGI genes are functionally classified for the biosynthesis of amino acids (30 genes), cofactors and carriers (25 genes), nucleotides (20 genes), and metabolic intermediates (15 genes) (Figure 2).

To further interrogate the VGI dataset, two previously uncharacterized genes, RL0920 a putative ATP-binding mrp family protein and RL3335 a putative lysophospholipase, were selected for targeted mutagenesis. A growth curve in liquid culture was used to characterize the generation time and growth response of the mutants. As expected, MA0920 and MA3335 were substantially growth impaired with increased generation times in VMM-mannitol. After 72 h of growth the mutant cultures were 1/3 the density of the wildtype in VMM-mannitol, whereas in TY they had achieved similar densities to the wildtype (Figure 3).

The TGI genes were composed of the smallest number of GI genes and the least functional complexity (Figure 2). Unlike the VGI genes, the general mechanism underlying the TGI genes does not hinge on metabolic biosynthesis, which is not surprising as complex media will contain many, if not all, metabolic intermediates required for growth. The largest functional categories observed in the TGI genes were hypothetical proteins (20 genes), cell envelope (11 genes), macromolecule synthesis and modification (9 genes), and transport and binding proteins (8 genes). Previous studies have identified several TGI genes, which collectively are implicated in outer membrane integrity or periplasmic function, suggesting that growth on complex media may require specific envelope traits (Gilbert et al., 2007; Vanderlinde et al., 2009, 2011; Foreman et al., 2010; Vanderlinde and Yost, 2012a,b). Additionally, we observed a putative cold shock response protein (RL2964), a LysR family transcriptional regulator, multiple transcriptional response regulators from predicted two-component systems (RL0036, RL1433, RL1729), a histidine kinase component of a two-component system (RL1382), as well as hypothetical proteins containing transmembrane domains (RL1526, RL2641), AAA–ATPase domain (RL2625), or N-terminal secretion signals (RL3761, RL1528, RL1618A, RL2086, RL4716) that were GI on TY medium (Supplementary File 1). These findings suggest that the TGI phenotype collectively may be centered around cell sensing and stress response at the cell envelope-environment interface.

### Central carbon metabolism of mannitol

Using INSeq we were able to screen the 4 major conserved central carbon metabolic pathways for genes required for growth on mannitol (Figure 4; Table 2). As expected, disruption of the genes required for mannitol transport and conversion to F6P, along with glucose-6-phosphate isomerase (*pgi*) required for conversion of F6P into glucose-6P (Keele et al., 1969; Arias et al., 1979), resulted in a GI phenotype. Genes required for conversion of G6P into gluconate-6P (GN6P) through the ED pathway (overlapping the oxidative branch of the pentose phosphate pathway) resulted in a GI phenotype when mutated on both TY and VMM (Figure 4: reaction 1.1–2). The essential nature of these reactions may be due to several factors: (1) the NADPH generated during conversion (Spaans et al., 2015), (2) the possibly toxic accumulation of phosphorylated intermediates (Cerveñanský and Arias, 1984; Kadner et al., 1992), (3) the role of G6P in the biosynthesis of osmoprotectants (Barra et al., 2003), and (4) the need for carbon flux into the ED pathway for glycolytic growth (Arias et al., 1979; Glenn et al., 1984; Stowers, 1985). Conversion of GN6P into pyruvate through the ED pathway was determined to be VGI (Figure 4: reaction 1.3–4). Mutations in the upper EMP pathway, aside from *pgi*, were observed to be neutral, which is in agreement with previous work (Glenn et al., 1984). The lower EMP pathway (sometimes considered shared by the ED pathway), converts glyceraldehyde-3P (GA3P) into pyruvate, with mutants at all enzymatic steps appearing GI on mannitol, and at some steps on TY as well (Figure 4: reaction 2.5–9). The VGI nature of the lower EMP when grown on mannitol is possibly due to mutants being unable to metabolize GA3P produced from the ED pathway into the amino acid precursors glycerate-3P (G3P) or phosphoenolpyruvate (PEP), and as well catabolize GA3P into pyruvate (Finan et al., 1988) for use in the TCA cycle. The overall GI phenotype of mutants in the entire ED pathway, in contrast to the EMP pathway, suggests that it is the central pathway for glycolytic conversion of carbon into the central carbon intermediate pyruvate. This is in agreement with previous research indicating the ED pathway to be the preferred route of carbon metabolism for glycolytic growth of rhizobia and closely related genera (Stowers, 1985; Fuhrer et al., 2005; Geddes and Oresnik, 2014).

Conversion of pyruvate into the TCA cycle was observed to have 2 unique GI pathways (Figure 4: reaction 3.1–14). Conversion of pyruvate into the TCA through acetyl-CoA as an intermediate is a less direct route than the direct conversion of pyruvate into oxaloacetate, and mutants in pyruvate carboxylase (Figure 4: reaction 3.14) were observed to be more severally GI than in citrate synthase (Figure 4: reaction 3.2). Additionally, on TY media mutants in pyruvate dehydrogenase appeared GI, while mutants in pyruvate carboxylase were not. These findings are in agreement with anaplerotic production of oxaloacetate (OAA), via *pyc*-mediated fixation of CO_2,_ being important for replenishing OAA pools under minimal growth conditions (Gokarn et al., 2001; Sirithanakorn et al., 2014).

When grown on mannitol as a sole carbon source, mutants in TCA cycle genes were all observed to be GI, aside from the conversion from fumarate to malate (Figure 4: reaction 3.2–3.9). Mutants in *fumC* and *fumA* (RL2701 and RL2703) were observed to be growth neutral, possibly due to functional redundancy between the two isozymes, which has been previously reported in *Bradyrhizobium japonicum* (Acuña et al., 1991). Future INSeq studies with prolonged exposure to selective pressure may identify which isoenzyme is dominant. Several TCA steps were observed to be GI on both VMM-mannitol and TY media, confirming that the TCA cycle is an important component of the CFG in RLV3841. Mutants in isocitrate dehydrogenase (*icd*) were both VGI and TGI (Figure 4: reaction 3.4). It has been previously shown that mutants in *icd* develop glutamate auxotrophy (McDermott and Kahn, 1992), which may explain the impaired growth phenotype on minimal medium, but does not explain growth impairment on peptide rich TY medium. Mutants in *sucB* and *citM* (*sucA*) were also observed to be growth impaired on both media (Figure 4: reaction 3.5). For growth on VMM-mannitol, the GI nature of these mutants is possibly due to increased α-ketoglutarate concentration due to the inability to metabolize into succinyl-CoA, and possibly perturbation of the GOGAT cycle via shunting of excess α-ketoglutarate (Bravo and Mora, 1988; Dunn, 1998).

The genes encoding enzymes for the glyoxalate by-pass, phosphoenolpyruvate carboxykinase and fructose bisphosphate aldolase, were observed to be GN on VMM-mannitol and TY media; suggesting that gluconeogenesis is not be required for growth in either condition (Kornberg, 1966; McKay et al., 1985; Stowers, 1985). This seems reasonable, as growth on mannitol is presumably glycolytic, therefore sugar conversion can be performed on metabolic intermediates generated during the breakdown of mannitol. And on TY media, many carbohydrates and polyols are likely already present in trace amounts, mitigating the need for gluconeogenesis.

Almost every gene involved in the non-oxidative branch of the PP pathways was observed to have a neutral impact on growth when mutated. The only two reactions in the PP pathway that were GI on mannitol were for the conversion of ribulose-5P into ribose-5P, or alternatively xylulose-5P (Figure 4: reaction 4.2 and 4.3). The mutation of ribose-5-phosphate isomerase A appearing GI is logical as ribose-5P is a precursor for 5-phosphoriboosyl-α-1-pyrophosphate, which is the branching point for flux of carbon in nucleotide, histidine, nicotinamide, and tryptophan biosynthesis (Kilstrup et al., 2005; Switzer, 2009). The conversion of GN6P into ribulose-5P however was observed to be GN. There are two possible explanations for mutants in this step appearing GN: (1) functional redundancy in isozymes *Gnt* and *GntZ* compensates for mutation of either (Figure 4: reaction 4.1), or (2) ribulose-5P can be replenished from xylulose-5P derived from either F6P and G3P being shunted into the PP pathway (Figure 4: reaction 4.4–6) or the phosphoketolase pathway (EC 4.1.2.9). In general, the interconnectedness of the PP pathway makes it difficult to study single gene knock-outs, as mutants may adapt to interrupted pathways by using alternative metabolic routes or isozymes (Geddes and Oresnik, 2014).

### Plasmid growth impaired genes

There are unique opportunities and challenges for exploring plasmid biology when conducting INSeq experiments on bacterial species with genomes containing multiple large plasmids. Mutations that result in the loss of a plasmid from the accessory genome, due to impaired plasmid stability, will appear phenotypically identical to a GI mutant lost from the mutant pool due to a decreased growth rate. Therefore, it cannot be concluded directly from the INSeq data if a particular transposon insertion within a plasmid resulted in a GI phenotype, or instead compromised plasmid stability or replication. All plasmids contained a three gene cluster of *rep* genes that were observed to be essential (PGI) when mutated (Figure 5; Supplementary File 1), which in conjunction with the annotated function of these genes suggests the loss of these Tn insertion tags from the mutant population was due to impaired plasmid replication.

Beyond identifying putative *rep* genes, INSeq can be useful in identifying plasmid genes that provide the host cell with growth benefits under specific conditions. For example, pRL11 contains a putative 8 gene operon (pRL110625-32) predicted to be involved in cobalamin biosynthesis, that was severely PGI on VMM-mannitol, and moderately PGI on TY when mutated. Previous studies in *R. etli* identified similar growth phenotypes when a homologous cobalamin biosynthetic cluster on p42e was deleted (Landeta et al., 2011). Additionally, pRL120209 (putative *tpiA*) and pRL120210 (putative *rpiB*), which encode enzymes predicted to function in central carbon metabolism, were found to be PGI when grown on mannitol as a sole carbon source. Their chromosomal homologs RL2513 (*tpiA*) and RL2547 (*rpiB*) were both observed to be GN (Table 2). In a closely related *R. leguminosarum* bv. *viciae* strain VF39, the homologs of pRL120209 and pRL120210 are required for growth on erythritol as a sole carbon source (Yost et al., 2006). This suggests that pRL12 may carry genes important for the normal growth of RLV3841 under specific conditions, which is in agreement with previous studies that showed pRL12 cured strains of *R. leguminosarum* were unable to grow on minimal media (Hynes et al., 1989).

### Technical considerations of INSeq in RLV3841

INSeq, like all high-throughput molecular techniques, is not without limitations. Genes with large regions of sequence duplication, or no mariner “TA” insertion sites, cannot be assayed using INSeq. These genes represent only 1.9% of the genome. However, the targeted library preparation method and robust statistical analysis afforded by the use of MmeI-adapted *mariner* transposon appears to outweigh its disadvantages. A sufficient saturation of neutral mariner insertion sites within the mutant community allows for confident identification of regions that lack insertions due to the negative selection resulting from a GI phenotype. In this and previous, work a sufficient level of neutral “TA” site saturation has been recovered to allow Bayesian methods of analysis, using a relatively modest amount of sequencing data when compared to other *mariner* INSeq studies. Considerations in inoculation density, the number of generations of growth during negative selection, and the ability to recover mutant populations needs to be carefully considered in order to ensure enough complexity is retained in the mutant pools post-selection to allow for statistical analysis.

## Author contributions

BP and CY conceived and designed the research; BP and MA conducted the experiments; BP conducted the data analysis; BP and CY prepared and finalized the manuscript.

## Funding

The presented research was conducted with support from a Discovery Grant awarded by the Natural Sciences and Engineering Council of Canada.

### Conflict of interest statement

The authors declare that the research was conducted in the absence of any commercial or financial relationships that could be construed as a potential conflict of interest.

## References

[B1] Acuña G. Ebeling S. Hennecke H. (1991). Cloning, sequencing, and mutational analysis of the *Bradyrhizobium japonium fumC*-like gene: evidence for the exsistence of two different fumarases. J. Gen. Microbiol. 137, 991–1000. 10.1099/00221287-137-4-991 1856685

[B2] Arias A. Cervenansky C. Gardiol A. Martinez-Drets G. (1979). Phosphoglucose isomerase mutants of *Rhizobium meliloti*. J. Bacteriol. 137, 409–414. 762017 10.1128/jb.137.1.409-414.1979 PMC366610

[B3] Barquist L. Boinett C. J. Cain A. K. (2013). Approaches to querying bacterial genomes with transposon-insertion sequencing. RNA Biol. 10, 1161–1169. 10.4161/rna.24765 23635712 PMC3849164

[B4] Barra L. Pica N. Gouffi K. Walker G. C. Blanco C. Trautwetter A. (2003). Glucose 6-phosphate dehydrogenase is required for sucrose and trehalose to be efficient osmoprotectants in *Sinorhizobium meliloti*. FEMS Microbiol. Lett. 229, 183–188. 10.1016/S0378-1097(03)00819-X 14680697

[B5] Bishop A. H. Rachwal P. A. Vaid A. (2014). Identification of genes required by *Bacillus thuringiensis* for survival in soil by transposon-directed insertion site sequencing. Curr. Microbiol. 68, 477–485. 10.1007/s00284-013-0502-7 24310935

[B6] Bravo A. Mora J. (1988). Ammonium assimilation in *Rhizobium phaseoli* by the glutamine synthetase-glutamate synthase pathway. J. Bacteriol. 170, 980–984. 10.1128/jb.170.2.980-984.1988 2892829 PMC210751

[B7] Brutinel E. D. Gralnick J. A. (2012). Anomalies of the anaerobic tricarboxylic acid cycle in *Shewanella oneidensis* revealed by Tn-seq. Mol. Microbiol. 86, 273–283. 10.1111/j.1365-2958.2012.08196.x 22925268

[B8] Byrne R. T. Chen S. H. Wood E. A. Cabot E. L. Cox M. M. (2014). *Escherichia coli* genes and pathways involved in surviving extreme exposure to ionizing radiation. J. Bacteriol. 196, 3534–3545. 10.1128/JB.01589-14 25049088 PMC4187691

[B9] Capel E. Zomer A. L. Nussbaumer T. Bole C. Izac B. Frapy E.. (2016). Comprehensive identification of *Meningococcal* genes and small noncoding RNAs required for host cell colonization. MBio 7, e01173–e01116. 10.1128/mbio.01173-16 27486197 PMC4981724

[B10] Cerveñanský C. Arias A. (1984). Glucose-6-phosphate dehydrogenase deficiency in pleiotropic carbohydrate-negative mutant strains of *Rhizobium meliloti*. J. Bacteriol. 160, 1027–1030. 6501224 10.1128/jb.160.3.1027-1030.1984 PMC215813

[B11] DeJesus M. A. Ioerger T. R. (2013). A hidden Markov model for identifying essential and growth-defect regions in bacterial genomes from transposon insertion sequencing data. BMC Bioinformatics 14:303. 10.1186/1471-2105-14-303 24103077 PMC3854130

[B12] Dong T. G. Ho B. T. Yoder-Himes D. R. Mekalanos J. J. (2013). Identification of T6SS-dependent effector and immunity proteins by Tn-seq in *Vibrio cholerae*. Proc. Natl. Acad. Sci. U.S.A. 110, 2623–2628. 10.1073/pnas.1222783110 23362380 PMC3574944

[B13] Doyle J. J. Luckow M. A. (2003). The rest of the iceberg. Legume diversity and evolution in a phylogenetic context. Plant Physiol. 131, 900–910. 10.1104/pp.102.018150 12644643 PMC1540290

[B14] Dunn M. F. (1998). Tricarboxylic acid cycle and anaplerotic enzymes in rhizobia. FEMS Microbiol. Rev. 22, 105–123. 10.1111/j.1574-6976.1998.tb00363.x 9729766

[B15] Finan T. M. Oresnik I. Bottacin A. (1988). Mutants of *Rhizobium meliloti* defective in succinate metabolism. J. Bacteriol. 170, 3396–3403. 10.1128/jb.170.8.3396-3403.1988 2841284 PMC211307

[B16] Foreman D. L. Vanderlinde E. M. Bay D. C. Yost C. K. (2010). Characterization of a gene family of outer membrane proteins (*ropB*) in *Rhizobium leguminosarum* bv. *viciae* VF39SM and the role of the sensor kinase ChvG in their regulation. J. Bacteriol. 192, 975–983. 10.1128/JB.01140-09 20023026 PMC2812955

[B17] Fuhrer T. Fischer E. Sauer U. (2005). Experimental identification and quantification of glucose metabolism in seven bacterial species. Society 187, 1581–1590. 10.1128/jb.187.5.1581-1590.2005 15716428 PMC1064017

[B18] Gallagher L. A. Shendure J. Manoil C. (2011). Genome-scale identification of resistance functions in *Pseudomonas aeruginosa* using Tn-seq. MBio 2:e00315. 10.1128/mBio.00315-10 21253457 PMC3023915

[B19] Gawronski J. D. Wong S. M. S. Giannoukos G. Ward D. V. Akerley B. J. (2009). Tracking insertion mutants within libraries by deep sequencing and a genome-wide screen for *Haemophilus* genes required in the lung. Proc. Natl. Acad. Sci. U.S.A. 106, 16422–16427. 10.1073/pnas.0906627106 19805314 PMC2752563

[B20] Geddes B. A. Oresnik I. J. (2014). Physiology, genetics, and biochemistry of carbon metabolism in the alphaproteobacterium *Sinorhizobium meliloti*. Can. J. Microbiol. 60, 491–507. 10.1139/cjm-2014-0306 25093748

[B21] Gilbert K. B. Vanderlinde E. M. Yost C. K. (2007). Mutagenesis of the carboxy terminal protease CtpA decreases desiccation tolerance in *Rhizobium leguminosarum*. FEMS Microbiol. Lett. 272, 65–74. 10.1111/j.1574-6968.2007.00735.x 17456188

[B22] Glenn A. R. McKay I. A. Arwas R. Dilworth M. J. (1984). Sugar metabolism and the symbiotic properties of carbohydrate mutants of *Rhizobium leguminosarum*. Microbiology 130, 239–245. 10.1099/00221287-130-2-239

[B23] Gokarn R. R. Evans J. D. Walker J. R. Martin S. A. Eiteman M. A. Altman E. (2001). The physiological effects and metabolic alterations caused by expression of *Rhizobium etli* pyruvate carboxylase in *Escherichia coli*. Appl. Microbiol. Biotechnol. 56, 188–195. 10.1007/s002530100661 11499929

[B24] Goodman A. L. McNulty N. P. Zhao Y. Leip D. Mitra R. D. Lozupone C. A.. (2009). Identifying genetic determinants needed to establish a human gut symbiont in its habitat. Cell Host Microbe 6, 279–289. 10.1016/j.chom.2009.08.003 19748469 PMC2895552

[B25] Goodman A. L. Wu M. Gordon J. I. (2011). Identifying microbial fitness determinants by insertion sequencing using genome-wide transposon mutant libraries. Nat. Protoc. 6, 1969–1980. 10.1038/nprot.2011.417 22094732 PMC3310428

[B26] Griffin J. E. Gawronski J. D. Dejesus M. A. Ioerger T. R. Akerley B. J. Sassetti C. M. (2011). High-resolution phenotypic profiling defines genes essential for mycobacterial growth and cholesterol catabolism. PLoS Pathog. 7:e1002251. 10.1371/journal.ppat.1002251 21980284 PMC3182942

[B27] Gutierrez M. G. Yoder-Himes D. R. Warawa J. M. (2015). Comprehensive identification of virulence factors required for respiratory melioidosis using Tn-seq mutagenesis. Front. Cell. Infect. Microbiol. 5:78. 10.3389/fcimb.2015.00078 26583079 PMC4631991

[B28] Hooven T. A. Catomeris A. J. Akabas L. H. Randis T. M. Maskell D. J. Peters S. E.. (2016). The essential genome of *Streptococcus agalactiae*. BMC Genomics 17:406. 10.1186/s12864-016-2741-z 27229469 PMC4881062

[B29] Hynes M. F. Quandt J. O'Connell M. P. Pühler A. (1989). Direct selection for curing and deletion of *Rhizobium* plasmids using transposons carrying the *Bacillus subtilis sacB* gene. Gene 78, 111–120. 10.1016/0378-1119(89)90319-3 2548927

[B30] Johnson J. G. Livny J. DiRita V. J. (2014). High-throughput sequencing of *Campylobacter jejuni* insertion mutant libraries reveals *mapA* as a fitness factor for chicken colonization. J. Bacteriol. 196, 1958–1967. 10.1128/JB.01395-13 24633877 PMC4010991

[B31] Johnston A. W. B. Beringer J. E. (1975). Identification of *Rhizobium* strains in pea root nodules using genetic markers. J. Gen. Microbiol. 87, 343–350. 10.1099/00221287-87-2-343 1141859

[B32] Kadner R. J. Murphy G. P. Stephens C. M. (1992). Two mechanisms for growth inhibition by elevated transport of sugar phosphates in *Escherichia coli*. J. Gen. Microbiol. 138, 2007–2014. 10.1099/00221287-138-10-2007 1479338

[B33] Kamp H. D. Patimalla-Dipali B. Lazinski D. W. Wallace-Gadsden F. Camilli A. (2013). Gene fitness landscapes of *Vibrio cholerae* at important stages of its life cycle. PLoS Pathog. 9:e1003800. 10.1371/journal.ppat.1003800 24385900 PMC3873450

[B34] Keele B. B. Jr. Hamilton P. B. Elkan G. H. (1969). Glucose catabolism in *Rhizobium japonicum*. J. Bacteriol. 97, 1184–1191. 5776525 10.1128/jb.97.3.1184-1191.1969 PMC249833

[B35] Khatiwara A. Jiang T. Sung S. S. Dawoud T. (2012). Genome scanning for conditionally essential genes in *Salmonella enterica* serotype Typhimurium. Appl. Environ. Microbiol. 78, 3098–3107. 10.1128/aem.06865-11 22367088 PMC3346488

[B36] Kilstrup M. Hammer K. Ruhdal Jensen P. Martinussen J. (2005). Nucleotide metabolism and its control in lactic acid bacteria. FEMS Microbiol. Rev. 29, 555–590. 10.1016/j.fmrre.2005.04.006 15935511

[B37] Kornberg H. L. (1966). The role and control of the glyoxylate cycle in *Escherichia coli*. Biochem. J. 99, 1–11. 10.1042/bj0990001 5337756 PMC1264949

[B38] Kuehl J. V. Price M. N. Ray J. Wetmore K. M. Esquivel Z. Kazakov A. E.. (2014). Functional genomics with a comprehensive library of transposon mutants for the sulfate-reducing bacterium *Desulfovibrio alaskensis* G20. MBio 5, 1–13. 10.1128/mBio.01041-14 24865553 PMC4045070

[B39] Landeta C. Dávalos A. Cevallos M. Á. Geiger O. Brom S. Romero D. (2011). Plasmids with a chromosome-like role in rhizobia. J. Bacteriol. 193, 1317–1326. 10.1128/JB.01184-10 21217003 PMC3067620

[B40] Langridge G. C. Phan M.-D. Turner D. J. Perkins T. T. Parts L. Haase J.. (2009). Simultaneous assay of every *Salmonella* Typhi gene using one million transposon mutants. Genome Res. 19, 2308–2316. 10.1101/gr.097097.109 19826075 PMC2792183

[B41] Le Breton Y. Belew A. T. Valdes K. M. Islam E. Curry P. Tettelin H.. (2015). Essential genes in the core genome of the human pathogen *Streptococcus pyogens*. Sci. Rep. 5:9838. 10.1038/srep09838 25996237 PMC4440532

[B42] Lee S. A. Gallagher L. A. Thongdee M. Staudinger B. J. Lippman S. Singh P. K.. (2015). General and condition-specific essential functions of *Pseudomonas aeruginosa*. Proc. Natl. Acad. Sci. U.S.A. 112, 5189–5194. 10.1073/pnas.1422186112 25848053 PMC4413342

[B43] McDermott T. R. Kahn M. L. (1992). Cloning and mutagenesis of the *Rhizobium meliloti* isocitrate dehydrogenase gene. J. Bacteriol. 174, 4790–4797. 10.1128/jb.174.14.4790-4797.1992 1320616 PMC206277

[B44] McKay I. A. Glenn A. R. Dilworth M. J. (1985). Gluconeogenesis in *Rhizobium leguminosarum* MNF3841. Microbiology 131, 2067–2073. 10.1099/00221287-131-8-2067

[B45] Meeske A. J. Rodrigues C. D. A. Bradly J. Lim H. C. Bernhardt T. G. Rudner D. Z. (2015). High-throughput genetic screens identify a large and diverse collection of new sporulation genes in *Bacillus subtilis*. PLoS Biol. 14:e1002341. 10.1371/journal.pbio.1002341 26735940 PMC4703394

[B46] Moule M. G. Spink N. Willcocks S. Lim J. Guerra-Assunção J. A. Cia F.. (2015). Characterization of new virulence factors involved in the intracellular growth and survival of *Burkholderia pseudomallei*. Infect. Immun. 84, 701–710. 10.1128/IAI.01102-15 26712202 PMC4771355

[B47] Murray J. L. Kwon T. Marcotte E. M. Whiteley M. (2015). Intrinsic antimicrobial resistance determinants in the superbug *Pseudomonas aeruginosa*. MBio 6, e01603–e01615. 10.1128/mBio.01603-15 26507235 PMC4626858

[B48] Oldroyd G. E. D. Murray J. D. Poole P. S. Downie J. A. (2011). The rules of engagement in the legume-rhizobial symbiosis. Annu. Rev. Genet. 45, 119–144. 10.1146/annurev-genet-110410-132549 21838550

[B49] Pechter K. B. Gallagher L. Pyles H. Manoil C. S. Harwood C. S. (2015). Essential genome of the metabolically versatile Alphaproteobacterium *Rhodopseudomonas palestris*. J. Bacteriol. 198, 867–876. 10.1128/JB.00771-15 26712940 PMC4810602

[B50] Perry B. J. Yost C. K. (2014). Construction of a mariner-based transposon vector for use in insertion sequence mutagenesis in selected members of the *Rhizobiaceae*. BMC Microbiol. 14:298. 10.1186/s12866-014-0298-z 25433486 PMC4255674

[B51] Phan M. D. Peters K. M. Sarkar S. Lukowski S. W. Allsopp L. P. Gomes Moriel D.. (2013). The serum resistome of a globally disseminated multidrug resistant uropathogenic *Escherichia coli* clone. PLoS Genet. 9:e1003834. 10.1371/journal.pgen.1003834 24098145 PMC3789825

[B52] Quandt J. Hynes M. F. (1993). Versatile suicide vector which allows direct selection for gene replacement in gram-negative bacteria. Gene 127, 15–21. 10.1016/0378-1119(93)90611-6 8486283

[B53] Rubin B. E. Wetmore K. M. Price M. N. Diamon S. Shultzaberger R. K. Lowe L. C.. (2015)., The essential gene set of a photosynthetic organism. Proc. Natl. Acad. Sci. U.S.A. 112, E6634–E6643. 10.1073/pnas.1519220112 26508635 PMC4672817

[B54] Shan Y. Lazinski D. Rowe S. Camilli A. Lewis K. (2015). Genetic basis of persister tolerance to aminoglycosides in *Escherichia coli*. MBio 6, e00078–e00015. 10.1128/mBio.00078-15 25852159 PMC4453570

[B55] Sirithanakorn C. Adina-Zada A. Wallace J. C. Jitrapakdee S. Attwood P. V. (2014). Mechanisms of inhibition of *Rhizobium etli* pyruvate carboxylase by L-aspartate. Biochemistry 54, 7100–7106. 10.1021/bi501113u PMC4238798 25330457

[B56] Skurnik D. Roux D. Aschard H. Cattoir V. Yoder-Himes D. Lory S.. (2013). A comprehensive analysis of *in vitro* and *in vivo* genetic fitness of *Pseudomonas aeruginosa* using high-throughput sequencing of transposon libraries. PLoS Pathog. 9:e1003582. 10.1371/journal.ppat.1003582 24039572 PMC3764216

[B57] Spaans S. K. Weusthuis R. A. van der Oost J. Kengen S. W. M. (2015). NADPH-generating systems in bacteria and archaea. Front. Microbiol. 6:742. 10.3389/fmicb.2015.00742 26284036 PMC4518329

[B58] Stowers M. D. (1985). Carbon metabolism in *Rhizobium* species. Annu. Rev. Microbiol. 39, 89–108. 10.1146/annurev.mi.39.100185.000513 3904617

[B59] Switzer R. L. (2009). Discoveries in bacterial nucleotide metabolism. J. Biol. Chem. 284, 6585–6594. 10.1074/jbc.X800012200 18948259 PMC2652299

[B60] Tran T. Ran Q. Ostrer L. Khodursky A. (2016). *De novo* characterization of genes that contribute to high-level ciprofloxacin resistance in *Echerichia coli*. Antimicrob. Agents Chemother. 60, 6353–6355. 10.1128/AAC.00889-16 27431218 PMC5038283

[B61] Troy E. B. Lin T. Gao L. Lazinski D. W. Lundt M. Camilli A.. (2016). Global Tn-seq analysis of carbohydrate utilization and vertebrate infectivity of *Borrelia burgdorferi*. Mol. Microbiol. 101, 1003–1023. 10.1111/mmi.13437 27279039 PMC5028225

[B62] Turner K. H. Wessel A. K. Palmer G. C. Murray J. L. Whiteley M. (2015). Essential genome of *Pseudomonas aeruginosa* in cystic fibrosis sputum. Proc. Natl. Acad. Sci. U.S.A. 112, 4110–4115. 10.1073/pnas.1419677112 25775563 PMC4386324

[B63] Udvardi M. Poole P. S. (2013). Transport and metabolism in legume-rhizobia symbioses. Annu. Rev. Plant Biol. 64, 781–805. 10.1146/annurev-arplant-050312-120235 23451778

[B64] van Opijnen T. Bodi K. L. Camilli A. (2009). Tn-seq: high-throughput parallel sequencing for fitness and genetic interaction studies in microorganisms. Nat. Methods 6, 767–775. 10.1038/nmeth.1377 19767758 PMC2957483

[B65] van Opijnen T. Camilli A. (2013). Transposon insertion sequencing: a new tool for systems-level analysis of microorganisms. Nat. Rev. Microbiol. 11, 435–442. 10.1038/nrmicro3033 23712350 PMC3842022

[B66] Vanderlinde E. M. Harrison J. J. Muszynsji A. Carlson R. W. Turner R. J. Yost C. K. (2010). Identification of a novel ABC transporter required for desiccation tolerance, and biofilm formation in *Rhizobium leguminosarum* bv. viciae 3841. FEMS Microbiol. Ecol. 71, 327–40. 10.1111/j.1574-6941.2009.00824.x 20030718 PMC2868943

[B67] Vanderlinde E. M. Magnus S. A. Tambalo D. D. Koval S. F. Yost C. K. (2011). Mutation of a broadly conserved operon (RL3499-RL3502) from *Rhizobium leguminosarum* biovar viciae causes defects in cell morphology and envelope integrity. J. Bacteriol. 193, 2684–2694. 10.1128/JB.01456-10 21357485 PMC3133104

[B68] Vanderlinde E. M. Muszynski A. Harrison J. J. Koval S. F. Foreman D. L. Ceri H.. (2009). *Rhizobium leguminosarum* biovar viciae 3841, deficient in 27-hydroxyoctacosanoate-modified lipopolysaccharide, is impaired in desiccation tolerance, biofilm formation and motility. Microbiology 155, 3055–3069. 10.1099/mic.0.025031-0 19460825 PMC2850257

[B69] Vanderlinde E. M. Yost C. K. (2012a). Genetic analysis reveals links between lipid A structure and expression of the outer membrane protein gene, ropB, in *Rhizobium leguminosarum*. FEMS Microbiol. Lett. 335, 130–139. 10.1111/j.1574-6968.2012.02645.x 22845832

[B70] Vanderlinde E. M. Yost C. K. (2012b). Mutation of the sensor kinase chvG in *Rhizobium leguminosarum* negatively impacts cellular metabolism, outer membrane stability, and symbiosis. J. Bacteriol. 194, 768–777. 10.1128/JB.06357-11 22155778 PMC3272964

[B71] Verhagen L. M. de Jonge M. I. Burghout P. Schraa K. Spagnuolo L. Mennens S.. (2014). Genome-wide identification of genes essential for the survival of *Streptococcus pneumoniae* in human saliva. PLoS ONE 9:e89541. 10.1371/journal.pone.0089541 24586856 PMC3934895

[B72] Vincent J. M. (1970). A Manual for the Practical Study of Root-nodule Bacteria. IBP Handbook. Oxford: Blackwell Scientific.

[B73] Wang N. Ozer E. A. Mandel M. J. Hauser A. R. Hauser R. (2014). Genome-wide identification of *Acinetobacter baumannii* genes necessary for persistence in the lung. MBio 5, e01163–e01114. 10.1128/mBio.01163-14 24895306 PMC4049102

[B74] Wielbo J. (2012). Rhizobial communities in symbiosis with legumes: genetic diversity, competition and interactions with host plants. Cent. Eur. J. Biol. 7, 363–372. 10.2478/s11535-012-0032-5

[B75] Yang H. Krumholz E. W. Brutinel E. D. Palani N. P. Sadowsky M. J. Odlyzko A. M.. (2014). Genome-scale metabolic network validation of *Shewanella oneidensis* using transposon insertion frequency analysis. PLoS Comput. Biol. 10:e1003848. 10.1371/journal.pcbi.1003848 25233219 PMC4168976

[B76] Yost C. K. Rath A. M. Noel T. C. Hynes M. F. (2006). Characterization of genes involved in erythritol catabolism in *Rhizobium leguminosarum* bv. viciae. Microbiology 152, 2061–2074. 10.1099/mic.0.28938-0 16804181

[B77] Young J. P. Crossman L. C. Johnston A. W. (2006). The genome of *Rhizobium leguminosarum* has recognizable core and accessory components. Genome 7, R34. 10.1186/gb-2006-7-4-r34 16640791 PMC1557990

[B78] Yung M. C. Park D. M. Overton K. W. Blow M. J. Hoover C. A. Smit J.. (2015). Transposon mutagenesis paired with deep sequencing of *Caulobacter crescentus* under uranium stress reveals genes essential for detoxification and stress tolerance. J. Bacteriol. 197, 3160–3172. 10.1128/JB.00382-15 26195598 PMC4560278

